# Hancinone possesses potentials on increasing the ability of HMC3 cells to phagocytosis of Aβ_1-42_ via TREM2/Syk/PI3K/AKT/mTOR signaling pathway

**DOI:** 10.1371/journal.pone.0324202

**Published:** 2025-05-27

**Authors:** Yushun Zhou, Guran Yu, Hao Li

**Affiliations:** 1 Department of Neurology, Jiangsu Province Hospital of Chinese Medicine, The Affiliated Hospital of Nanjing University of Chinese Medicine, Nanjing, Jiangsu Province, China; 2 Wangjing Hospital, China Academy of Chinese Medical Sciences, Beijing, PR China; Noorda College of Osteopathic Medicine, UNITED STATES OF AMERICA

## Abstract

**Context:**

The amyloid hypothesis is the most widely accepted explanation for Alzheimer’s disease (AD). Failure of microglia Amyloid β-protein (1–42) (Aβ1–42) oligomer clearance and secondary neuroinflammation play a crucial role in the etiology in sporadic AD. *Piper kadsura* (Choisy) Ohwi (PkO), an herb of Chinese medicine, has anti-inflammation, antioxidation effects.

**Objective:**

To explore the impact of PkO and its active substances on Alzheimer’s disease.

**Materials and methods:**

We integrated drug prediction, network pharmacology and molecular docking techniques to systematically examine multi-scale mechanisms of PkO. Moreover, human Microglia Clone 3 (HMC3) were respectively incubated for 24 hours in the presence or absence of Syk inhibitor (SI, 100 nmol/L), β-amyloid (1-42) oligomer mixtures (called as Aβ oligomer hereafter, Aβ, 2.5 µM), or hancinone (HAN, 0.5 µM, 2.5 µM, 10 µM) to verify the target of the effect of PkO on Aβ oligomer-induced microglia.

**Results:**

Ultimately, we screened hancinone from PkO as a potential therapeutic agent for AD. Hancinone increased Triggering receptor expressed on myeloid cells 2 (TREM2), Syk, and p-Syk levels, up-regulated relative levels of p-PI3K, p-AKT, and mTOR, promoted the ability of HMC3 cells from the M1 phenotype to the M2 phenotype in Aβ or SI-stimulated HMC3 cells, and enhanced the phagocytic capacity of HMC3 cells to Aβ.

**Discussion and conclusions:**

Hancinone could regulate the phenotype of HMC3 cells and promote cell phagocytosis of Aβ by modulating the TREM2/Syk/PI3K/AKT/mTOR signaling pathway. This systematic exploration indicates that hancinone has the therapeutic effect on AD.

## Introduction

Alzheimer’s disease is a prevalent neurodegenerative disease that is characterized by a gradual decline in cognitive ability and impaired memory, coupled with alterations in personality and behavior [1]. Currently, there are more than 45 million people suffering from Alzheimer’s disease, mainly the elderly over 65 years old [2]. The mechanisms and causes of Alzheimer’s disease remain incompletely understood. Donepezil, a common cholinesterase inhibitor, is prescribed for the clinical treatment of Alzheimer’s disease. Although clinical trials indicate that donepezil, cholinesterase inhibitors, shows efficacy short term, it does not appear to mitigate the deteriorating clinical course of Alzheimer’s disease [3]. Recent failures in phase III clinical trials and limited progress in the development of therapeutic drugs indicate the pressing need to explore alternative approaches for treating Alzheimer’s disease (AD) [4].

Microglia, essential phagocytes of the central nervous system (CNS), play a vital role in the development, maintenance, and protection of the CNS [5]. Interestingly, the triggering receptor expressed on myeloid cell 2 (TREM2) is an innate immune receptor specifically expressed in microglia. Previous research has indicated that deficiency of TREM2 augments the accumulation of amyloid β and neuronal loss in a mouse model of Alzheimer’s disease [6]. The TREM2 activates microglia through the Syk-dependent pathway [7]. Since the TREM2 triggers microglia activation through the PI3K-AKT-mTOR signaling pathway, and the deficiency of Syk obstructs the activation of PI3K-AKT-GSK3β-mTOR axis [8], the Syk signaling has been characterized as a central node in microglia in response to Aβ pathology. Additionally, some natural ligands activating Syk signal have been detected to protect neurons, limit amyloid β and prevent AD progression [9].

The dry cane of *Piper kadsura* (Choisy) Ohwi (PkO), an herb plant used in traditional Chinese medicine (TCM) [10], contains several chemical compositions such as terpenoid, lignans and alkaloid [11]. Extracts from the PkO exhibit diverse biological functions, including anti-inflammatory, antioxidant, and neuroprotection [12–14]. However, the effect of the active constituents of PkO on microglia and TREM2/Syk/PI3K/AKT/mTOR signaling pathway remains unclear. In this research, we integrated drug prediction, network pharmacology and molecular docking techniques to systematically examine multi-scale mechanisms of PkO [15], investigated the therapeutic effects of PkO on Alzheimer’s disease (AD), identified a potential ligand of PkO that had therapeutic benefits on crucial AD and its targets.

## Materials and methods

### Data collection

#### Forecast of AD-related targets.

To conduct a search relevant to “Alzheimer’s disease,” three databases were utilized: DrugBank database (https://www.drugbank.ca/), GeneCards database (https://www.genecards.org/), and OMIM database (https://omim.org/). These databases were employed to ascertain targets associated with Alzheimer’s disease (AD). After removing the duplicate values from these three databases, the results were prospective AD-related genes.

#### Filtering active ingredients of PkO.

The active constituents of PkO were achieved from a reference-guided database of traditional Chinese medicine (S1 and S2 Table), utilizing a systems pharmacology-based approach with experimental validation. (TCMSP, http://old.tcmsp-e.com/) [10]. We utilized the SwissADME tool (http://swissadme.ch) to forecast plausible active constituents. The assessment encompassed the permeability of the blood-brain barrier (BBB), evaluation of gastrointestinal (GI) absorption, and conducted drug-likeness (DL) analysis [16]. The ADME screening mentioned the criteria defined by Lipinski’s rules, which include the following: the molecular weight should be less than or equal to 500 g/mol, the Moriguchi octanol-water partition coefficient should be less than or equal to 4.15, the number of nitrogen or oxygen atoms should be less than or equal to 10, and the number of NH or OH groups should be less than or equal to 5. All bioactive compounds fulfilled the requirements of the “Abbott Bioavailability Score (>0.1)” as determined by SwissADME. Typically, active ingredients encompass compounds that exhibit blood-brain barrier permeability, high gastrointestinal (GI) absorption, and conform to Lipinski’s rules during drug-likeness analysis [17,18]. Such compounds, once meeting these screening criteria, are identified as active ingredients possessing therapeutic efficacy.

#### Forecasting potential targets of active ingredients.

We used SwissTargetPrediction (http://www.swisstargetprediction.ch/) to fetch and forecast the associated targets of compounds in PkO. SwissTargetPrediction, a reputable database, leverages the 2D and 3D structures of established compounds for target prediction [19]. In this study, the target screening criterion was defined as a probability value exceeding zero.

#### Screening shared AD-related targets and performing visual analysis.

The intersection of the targets of Alzheimer’s disease and active ingredients was identified by Jvenn (https://jvenn.toulouse.inrae.fr/), an interactive Venn diagram viewer [20]. These shared targets are the estimated targets of the active ingredients of PkO for the treatment of AD. Cytoscape 3.7.2 software was utilized to construct, visualize, and analyze the network that connects herbs, active ingredients, and disease-related targets, with the aim of comprehending their intricate relationship.

#### GO and KEGG pathway enrichment analysis.

We utilized the R package from the “org.Hs.e.g.,db” database to select the estimated targets and used DOSE, clusterProfiler, and pathview package (Bioconductor) to perform GO enrichment analysis from three aspects, Biological Process (BP), Cellular Component (CC), and Molecular Function (MF). The top 10 results of each form were chosen to use bar charts or scatter diagrams for visual analysis. Based on the shared targets, we used DOSE, clusterProfiler, and pathview packages (Bioconductor) to conduct KEGG pathway enrichment analysis to forecast its mechanism of action using. Bar charts or scatter diagrams were conducted for visualization. The analysis was performed with P-value cutoff as 0.05 and Q-value cutoff as 0.05.

#### Protein-protein interaction data.

We imported the shared targets into the Search Tool for the Retrieval of Interacting Genes (STRING) database (https://string-db.org) and constructed a protein-protein interaction (PPI) network [21]. We chose “Homo sapiens” as the target-oriented species, selected the highest confidence scores (> 0.9) and deleted the isolated vertices. The measurement of the local centrality of a node in the network relied heavily on degree centrality, an essential parameter. In general, a node owning a high degree centrality might be the essential node of the network. In our study, we chose the top thirty targets in the PPI network regarding degree centrality as key targets for further research.

#### Data screening and visualization.

Based on the outcomes of GO and KEGG pathway enrichment analysis and PPI network, a compound-target-pathway map was constructed using Cytoscape 3.7.2 software to screen targets and pathways for verification in follow-up studies.

#### Molecular docking validation.

We conducted molecular docking with AutoDock and AutoDock Vina software which is based on molecular modeling techniques to forecast the directional interaction between the pivotal targets and compounds [22]. From Protein Data Bank (PDB, http://www.rcsb.org), we successfully achieved the crystal structure of the several objectives. We gained the structure of components from the PubChem database (https://pubchem.ncbi.nlm.nih.gov). Prior to conducting molecular docking experiments, we employed AutoDock software (version 4.2.6) for removing the ligand, hydrogenation, charge calculation, addition of protein type to macromolecules, and saving the resultant structures in PDBQT format. Following that, molecular docking between macromolecules and ligands was executed using AutoDock Vina, adhering to the predefined parameters. Binding energy of less than “-5” meant a grater combining interaction between macromolecules and small molecules [23]. In final, the PyMOL software (version 2.6.0) was utilized for the visualization of molecular docking.

### Experimental verification

#### Reagents and antibodies.

Hancinone (molecular weight 340.4; HPLC grade, > 97%) was purchased from Shanghai Acmec Biochemical Technology Co., Ltd. β-Amyloid (1–42) was purchased from GL Biochem Co., Ltd. 3-(4, 5-dimethyl-2-thiazolyl)-2, 5- diphenyl-2-H-tetrazolium bromide (MTT), and dimethyl sulfoxide (DMSO) were purchased from Sigma-Aldrich. R406 (Syk inhibitor) and Triton X-100, DAPI Staining Solution (C1005) were purchased from Beyotime. 0.25% of trypsin was from Keygen, Ltd. The 5-FITC-(Acp)-β-amyloid (1–42), human oligomer (sequence 5-FITC-(Acp)-DAEFRHDSGYEVHHQKLVFFAEDVGSNKGAIIGLMVGGVVIA; purity 96.17%) was purchased from ChinaPeptides Co., Ltd. RPMI 1640 was from Servicebio and fetal bovine serum (FBS) was purchased from Yuanyi Biotechnology Co., Ltd.

Phospho-Syk (Try525/526) antibody (Cat No: #2710), Syk antibody (Cat No: #13198) and phospho- AKT(Ser473) antibody (Cat No: #4060) were obtained from Cell Signaling Technology (Denver, USA). Pan-AKT antibody (Cat No: A18675), phospho-PI3K antibody (Cat No: AP0427), PI3 Kinase antibody (Cat No: A4992), β-actin antibody (Cat No: AC026) and HRP Goat Anti-Rabbit IgG (H + L) antibody (Cat No: AS014) were obtained from ABclonal Biotech Co., Ltd. TREM2 Polyclonal antibody (Cat No: AF09731) was from AiFang Biological Co., Ltd. mTOR antibody (Cat No: ab134903) was from Abcam. CD68 antibody was from Invitrogen. FITC CD206 (MMR) antibody (Cat No: 141703) was purchased from Biolegend (S3 Table).

The compound Hancinone was prepared by dissolving it in sterile deionized water at a concentration of 2 mM. This solution was then stored at a temperature of -80°C. When needed, the Hancinone solution was diluted to the desired concentrations using culturing medium. Syk inhibitor was dissolved in Phosphate Buffered Saline (PBS) at 40 µM, stored at 4°C, and diluted in culturing medium to desired concentrations immediately before use. We used an alternative approach to simulate Aβ formation because that *in vitro* experiments could not model complex Aβ production *in vivo*. Thus, we added sufficient chilled HFIP to the Aβ1–42 powder (GL Biochem) and mixed thoroughly to ensure complete dissolution of the powder. After incubating at room temperature for 60 min, the solution was placed on ice for 10 min before transferred to a centrifuge tube. We leaved the tube uncapped and placed it in a fume hood overnight at room temperature and used a freeze dryer to allow the HFIP to evaporate completely, leaving a clear and transparent film. We dissolved the film completely in DMSO and diluted it with F12 medium to prepare a final concentration of 250 µM Aβ solution (S2 Fig and S1 Report), maintaining sterile operations during the experiment to avoid contamination [24].

#### Cell culture and stimulation.

Human Microglia Clone 3 (HMC3) cells were purchased from Procell Life Science& Technology Co., Ltd. (Wuhan, China). The growth and maintenance of cells were in RPMI 1640 (Servicebio), supplemented with 20% heat-inactivated fetal bovine serum, 100 U/mL of penicillin, 100 μg/mL of streptomycin and 0.25 μg/mL of Amphotericin B (Bio-Channel). Cells were cultured at the temperature of 37°C and the environment of 5% CO2. Afterwards, cells were plated in 6-well plates until cells reached 80% confluence and then incubated with serum-free medium for another 6 h before Syk inhibitor, β-amyloid (1–42) oligomer, or hancinone supplementation. Cells were then incubated for 24 h in the presence or absence of Syk inhibitor (SI, 100 nmol/L), Aβ oligomer (Aβ, 2.5 µM), or hancinone (HAN, 0.5 µM, 2.5 µM, 10 µM) to estimate the influence of hancinone treatment on HMC3 responsiveness to analyze the impact of hancinone treatment on TREM2/Syk signaling.

#### Cell viability assay.

The 3-(4,5-dimethylthiazol-2-yl)-2,5-diphenyltetrazoliumbromide (MTT) assay is a sensitive survey of the regular metabolic condition of cells [25]. In this experiment, a solution of MTT (5 mg/mL) was added to each sample in a volume of 20 μL. Due to the presence of 180 μL of cell culture medium in each well, the final concentration of MTT is 0.5 mg/mL. MTT is an indirect measurement method that can be influenced by cell type and status, and MTT reagent possesses a certain degree of toxicity, which may have some impact on the experiment. During the experimental process, we refined the accuracy of the experimental results through careful operation. The purpose of this addition was to assess the viability of the cells. The samples were then incubated for 4 h at a temperature of 37°C and in the presence of 5% CO2. Following the incubation period, the culture medium was removed and the formazan products, which had formed because of the reaction with MTT, were dissolved in a volume of 150 μL DMSO each well. This step allowed for the quantification of the formazan products, which in turn provided a measure of cell viability. Each well was measured by light absorbance at 490 nm to calculate the appropriate concentration of Syk inhibitor, β-amyloid (1–42) oligomer, and hancinone for further study, which means that these three drugs did not exert toxic effects on HMC3 cells within the corresponding concentration ranges (S1 Fig).

#### Western blotting.

To calculate the appropriate concentration of SI and Aβ treated on HMC3 cells, the cells were respectively incubated with: SI group 1–6 (0, 25, 50, 100, 150, and 200 nmol/L SI), Aβ group 1–7 (0, 0.1, 0.25, 0.5, 0.75, 1 and 2.5 μmol/L Aβ). Further, according to the stimulation mentioned above, the cells were respectively incubated with control I (culture medium), model group I (100 nmol/L SI), low-concentration HAN group I (100 nmol/L SI + 0.5 μmol/L HAN), medium-concentration HAN group I (100 nmol/L SI + 2.5 μmol/L HAN), high-concentration HAN group I (100 nmol/L SI + 10 μmol/L HAN), control II (culture medium), model group II (2.5 μmol/L Aβ), low-concentration HAN group II (2.5 μmol/L Aβ + 0.5 μmol/L HAN), medium-concentration HAN group II (2.5 μmol/L Aβ + 2.5 μmol/L HAN) and high-concentration HAN group II (2.5 μmol/L Aβ + 10 μmol/L HAN). Following the administration of medication, the cells underwent two purges using ice-cold PBS (137 mM NaCl, 2.7 M KCl, 10 mM Na2HPO4, and 1.8 mM KH2PO4, pH 7.4). Subsequently, the cells were lysed using SDS sample buffer. Protein-equivalent cell lysates were subjected to SDS-PAGE (Beyotime) and subsequently transferred onto polyvinylidene difluoride (nitrocellulose) membranes. Each primary antibody was diluted using the primary antibody dilution buffer (Beyotime) according to the concentration specified in the corresponding instruction manual and the secondary antibody was diluted using the secondary antibody dilution buffer (Beyotime) at a ratio of 1:2000. Following a blocking step using blocking buffer (NCM Biotech), the membranes were incubated overnight with the suitable primary antibodies at 4°C. Finally, the membranes were exposed to secondary antibodies for 2 h at room temperature. We used the enhanced chemiluminescence system from Nanjing Vazyme Biotech Co., Ltd. to visualize immunoreactive proteins. We used the ratio of the target protein band intensity to the corresponding internal control protein band on the same membrane for subsequent calculations. Imaging and analysis with Image Lab software.

#### Flow cytometric analysis of phagocytosis and polarization.

Cells were harvested using EDTA-free trypsin solution (Beyotime). Following complete digestion neutralization, the cell suspension was transferred to a pre-chilled centrifuge tube and subjected to centrifugation at 1000 rpm for 5 min (4°C). After careful aspiration of the supernatant, the pellet was resuspended in PBS for gentle washing via pipette mixing, followed by repeated centrifugation under identical parameters. Then, cells were reconstituted in ice-cold FACS Buffer at a concentration of 1–5 × 10⁶ cells/mL for downstream applications. Afterwards, to analyze the phagocytic ability of HMC3 cells to Aβ, cells were stained by the 5-FITC-(Acp)-β-amyloid (1–42), human oligomer (QYAO Biotech) according to the manufacturer’s instructions. After 30-min incubation in the dark at 37°C incubator, cells were washed with 1-ml FACS buffer examined on a FACSCalibur (BD Biosciences) flow cytometer. In the research of polarization, cells were orderly stained by CD68 antibody (Invitrogen) for 30 min and CD206 (MMR) antibody (Biolegend) for 60 min in the dark at 37°C. Cells were then washed with 1-ml FACS buffer and then examined on a FACSCalibur (BD Biosciences) flow cytometer. According to the characteristics of the experimental subject HMC3 cells, corresponding FSC and SSC parameters are set to accurately identify HMC3 cells. Since the cells themselves do not contain stained Aβ oligomers, we could explore the amount of Aβ oligomers phagocytosed by HMC3 cells to a certain extent. We first used single-stain tubes for detection to eliminate interference. At the same time, based on the situation of singly stained cells, omissions or misidentifications are avoided, and further analysis is carried out after setting appropriate gating. The data obtained above were analyzed with FlowJo v10.8.1.

### Statistical analysis

Means ± standard deviation (S.D.) were used to express all outcomes in this study. Data were assessed using SPSS26.0, and the experiments were performed at least three times for each case. Initial analysis involved checking the normality of the data and analysis of variance (ANOVA), followed by conducting appropriate statistical tests. Since the results followed a normal distribution, a Student’s t-test was applied for simple comparisons, while a One-way ANOVA was used for multiple comparisons and a Tukey’s test was used for post-hoc multiple comparison test. Significance levels were reported as * P < 0.05, ** P < 0.01, *** P < 0.001.

## Results

### Candidate PkO targets for AD

**Fig 1:**
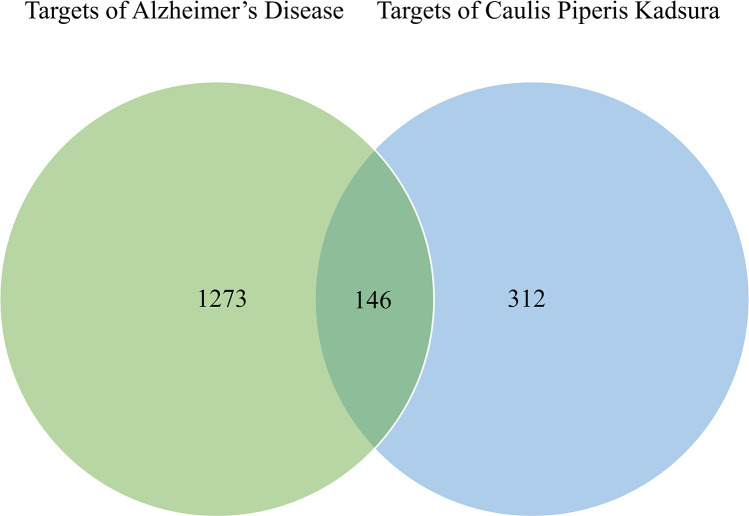
Venn diagram of overlapped targets between AD-related genes and targets from PkO. The green circle represents AD-related genes from DrugBank, GeneCards, and OMIM databases. The blue circle represents the targets from ingredients in PkO from TCMSP database.

**Fig 2:**
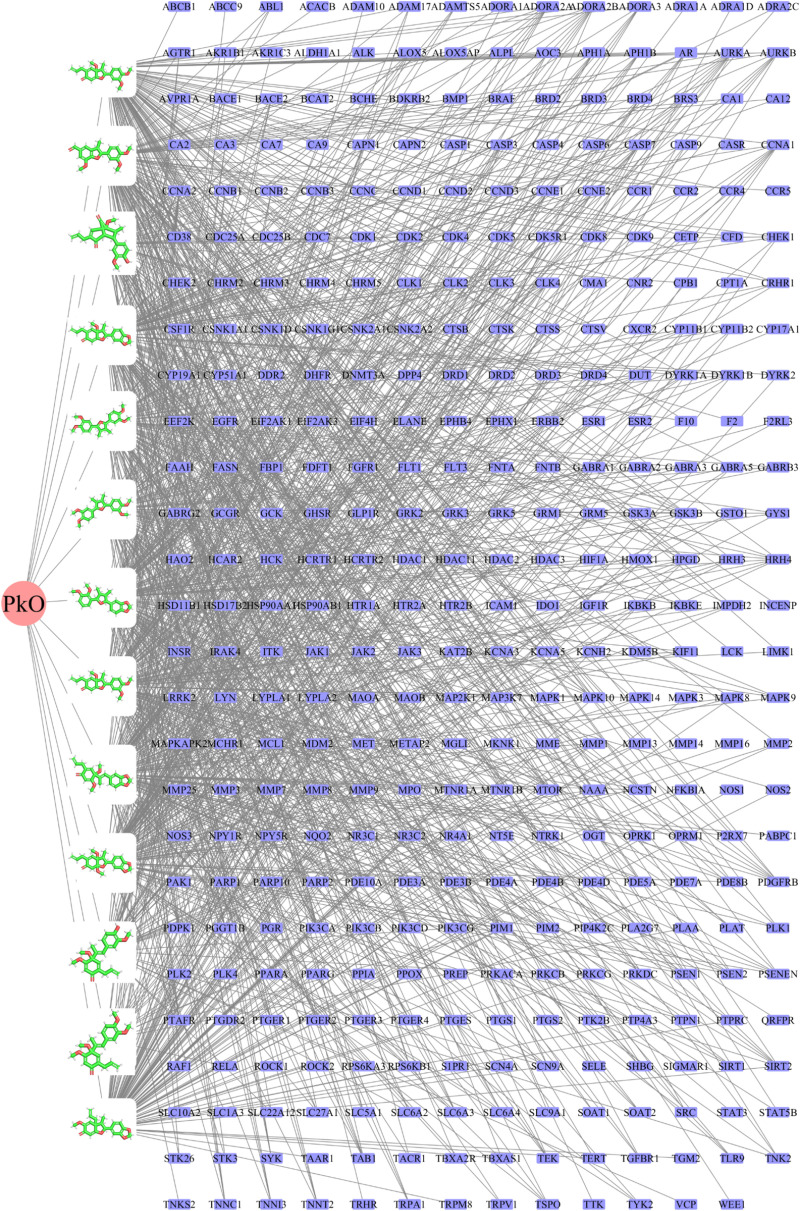
The network construction of PkO-ingredients-targets. The green node represents 13 active ingredients of PkO, and the blue node represents the predicted targets from these 13 active ingredients.

The DrugBank, GeneCards and OMIM databases were used to screen potential targets in AD. As a result, 88 AD-related targets were obtained from DrugBank, 908 AD-related targets were obtained from GeneCards, and 546 AD-related targets were gained from OMIM. 1419 potential targets related to AD were retained after the removal of duplicate values. In this research, we filter out 13 active ingredients of *PkO* using SwissADME. The GI absorption, BBB permeability, and TPSA (Topological Polar Surface Area) values of all bioactive compounds were also within acceptable limits (S1 Table). After removing duplicates, we utilized the Swiss Target Prediction database to import 13 unique active components. The database yielded a remarkable outcome of 458 potential targets. To extract targets pertinent to AD pathology, we created a Venn diagram. Consequently, we identified a noteworthy intersection of 146 targets (Fig 1). To provide a visual representation, we employed Cytoscape to establish a network consisting of 159 nodes (13 ingredients and 146 targets) and 1161 edges (Fig 2).

### Functional pathway of PkO targets

**Fig 3:**
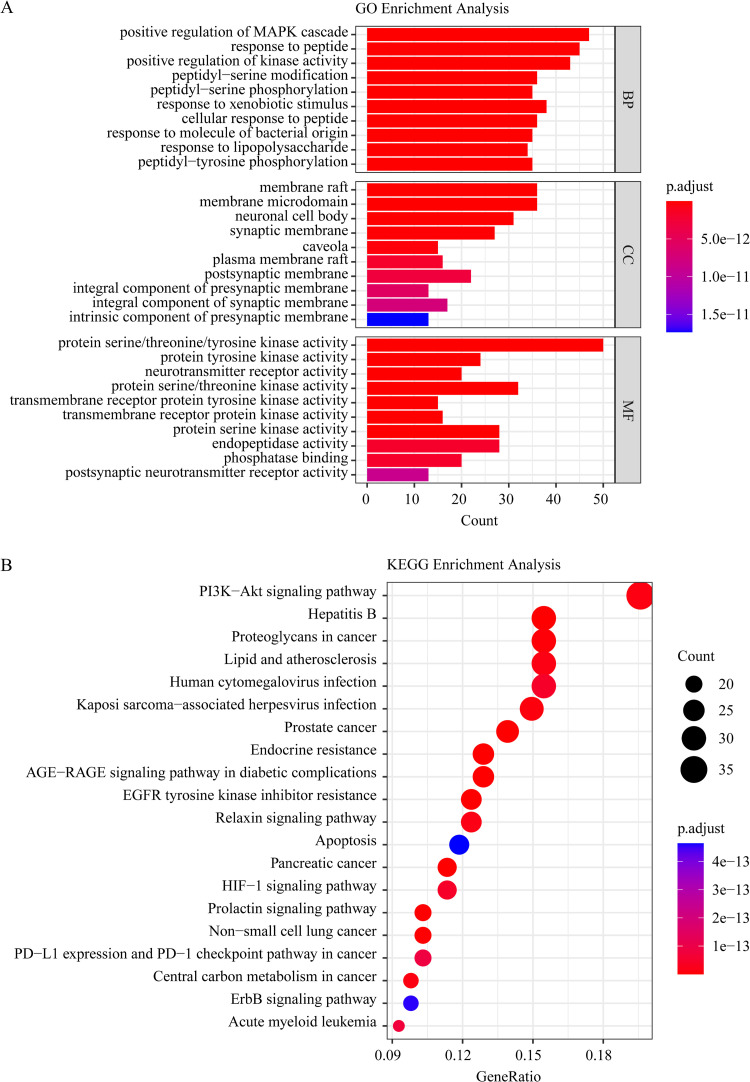
The GO and KEGG enrichment analysis of the common gene targets. A, Gene ontology terms of candidate targets of PkO against AD. The top 10 terms in each GO category with P Adjust Value < 0.05 were selected. BP, biological process; CC, cellular component; MF, molecular function. The axis Y designates the GO entry, and the square measure of the axis X and bar chart designate the figure of genes pertains to GO in candidate targets. B, KEGG pathway enrichment of candidate targets of PkO against AD. 20 terms with P Adjust Value < 0.05 and sorted by gene counts. The axis Y designates the name of pathways, axis X, and orbicular area designate the number of genes pertains to this signal pathway in candidate targets.

We conducted GO and KEGG enrichment analysis to uncover the essence of shared targets. The threshold for statistical significance was established at adjusted P-values of less than 0.05. 2721 GO enrichment outcomes were obtained in total, including 2406 BP (biological process), 117 CC (cellular component), and 198 MF (molecular function) terms. Then, the top 10 significant terms of BP, CC, and MF were used for visualization (Fig 3A). We acquired 175 pathways in total using KEGG pathway enrichment. The mapping process involved selecting the top 20 pathways, sorted in descending order based on gene counts (Fig 3B).

### Results of molecule docking

**Fig 4:**
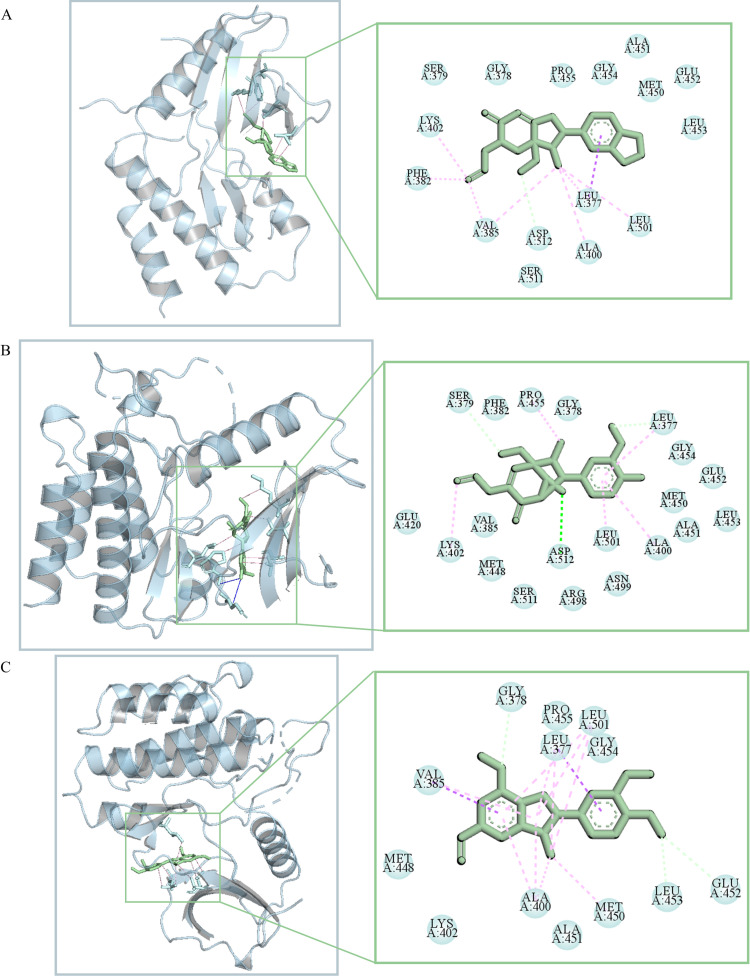
Three-dimensional view of the docking mode of three representative ingredients and Syk identified and conducted by Autodock and Pymol based on the model prediction. A, the docking mode of hancinone and Syk. The pink solid line represents hydrophobic interaction. B, the docking mode of kadsurenin k and Syk. The pink solid line represents hydrophobic interaction and the blue solid line represents hydrogen bonding interaction. C, the docking mode of kadsurenin m and Syk. The pink solid line represents hydrophobic interaction.

Based on the result of PkO targets for AD, KEGG, and GO enrichment analyses. Target Syk was selected for molecular docking with 3 representative ingredients associated with Syk and PI3K/AKT signaling pathway against AD. These top three docking combinations were MOL000315 and Syk, MOL000319 and Syk and MOL000321 and Syk, with energies of -8.1, -7.5, -7.9 kcal mol^-1^ respectively. We utilized PyMOL to display the three-dimensional view of the docking mode of three representative ingredients and Syk (Fig 4A–C). The results of ligand-receptor binding conformation indicated that hancinone has the best capacity of docking with Syk. Thus hancinone was chosen for further validation in subsequent experiments (S3 Fig).

### Functional pathway revalidation of Hancinone targets

**Fig 5:**
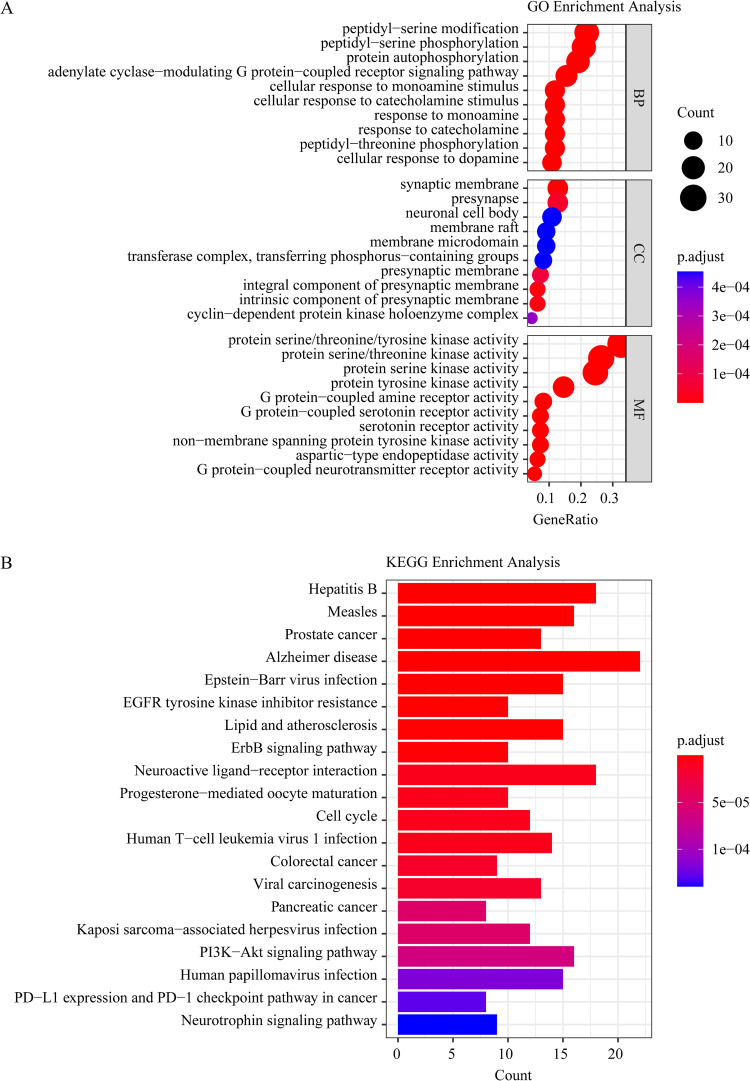
The GO and KEGG enrichment analysis of hancinone targets. A, Gene ontology terms of candidate targets of hancinone against AD. The top 10 terms in each GO category with P Adjust Value < 0.05 were chosen. BP, biological process; CC, cellular component; MF, molecular function. The axis Y designates the GO entry, and the square measure of the axis X and circular chart represent the number of genes pertaining to GO in candidate targets. B, KEGG pathway enrichment of candidate targets of hancinone against AD. 20 terms with P Adjust Value < 0.05 and sorted by gene counts. The axis Y designates the name of pathways, axis X, and bar area designate the number of genes pertains to this signal pathway in candidate targets.

In this study, GO and KEGG enrichment analyses were performed to further validate the functional substance of hancinone targets. We set the level of statistical significance at adjusted P-values < 0.05. 1142 GO enrichment outcomes were obtained in total, including 1005 BP (biological process) terms, 58 CC (cellular component) terms, and 79 MF (molecular function) terms. Then, the top 10 significant terms of BP, CC, and MF were used for visualization (Fig 5A). Through KEGG pathway enrichment, 132 enrichment pathways were obtained altogether. The mapping process involved selecting the top 20 pathways, sorted in descending order based on gene counts (Fig 5B).

### Constructing PPI network and core target verification

**Fig 6:**
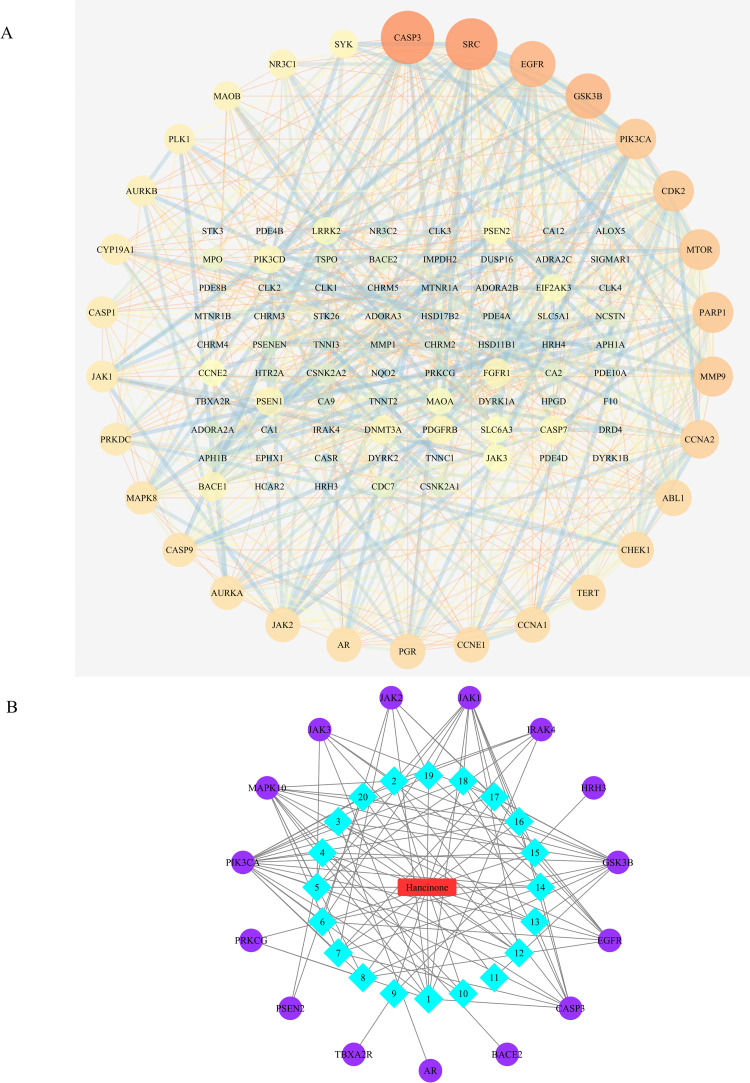
A, The primary PPI network of all overlapped targets of hancinone for treatment of AD by STRING database. Nodes represent proteins, and edges represent protein-protein associations. The top 30 relational targets arrange the circle layout on the periphery of the network. B, The network pharmacology approach to excavate the key 15 targets and 20 pathways in hancinone for further screening of 20 pathways and 15 genes related to hacinone and demonstration of correlations between them. The azure diamond node is the main pathway and the violet circular node is the key protein.

To further scope protein-protein interactions, the predicted targets of hancinone were analyzed by importing them into the STRING database. With a confidence threshold set at 0.9, a PPI network was acquired. After deleting the disconnected nodes and edges, we constructed the original PPI network (Fig 6A), which included 107 nodes and 585 edges. The interaction results were imported into Cytoscape 3.7.2 for network topology analysis to filter the top 30 targets arranged in descending order of degree centrality. The top 30 targets (CASP3, SRC, EGFR, GSK3B, PIK3CA, CDK2, MTOR, ARP1, MMP9, CCNA2, ABL1, CHEK1, TERT, CCNA1, CCNE1, PGR, AR, JAK2, AURKA, CASP9, MAPK8, PRKDC, JAK1, CASP1, CYP19A1, AURKB, PLK1, MAOB, NR3C1, SYK) were regarded as core targets, which included PI3K, MTOR and SYK. Although many targets among them are implicated in AD, JAK2 and MAPK are related to inflammation and CASP1 and CASP9 are tightly associated with cell apoptosis. In this study, we focus on phagocytosis-related functions thus PI3K, MTOR and SYK would be chosen for further research.

### Construction of a hancinone-pathway-target network

Based on the results of GO Analysis, and KEGG Analysis, Cytoscape3.7.2 was used to construct a hancinone-pathway-target network (Fig 6B). The network consisted of 15 targets, and 20 pathways (36 nodes and 116 edges). Then we used the built-in Network Analyzer tool to analyze the topological parameters of the network. The 20 pathways are linked to the selected AD targets. The 15 targets were received to their respective degrees: PIK3CA (degree = 18), MAPK10 (degree = 12), GSK3B (degree = 12), JAK1 (degree = 11), CASP3 (degree = 9), EGFR (degree = 8), JAK2 (degree = 6), JAK3 (degree = 6), IRAK4 (degree = 5), PRKCG (degree = 3), PSEN2 (degree = 2), AR (degree = 1), BACE2 (degree = 1), TBXA2R (degree = 1), and HRH3 (degree = 1).

### The viability of HMC3 cells treated with different concentrations of Syk inhibitor or β-amyloid (1–42) oligomer and the safety of hancinone (HAN)

**Fig 7:**
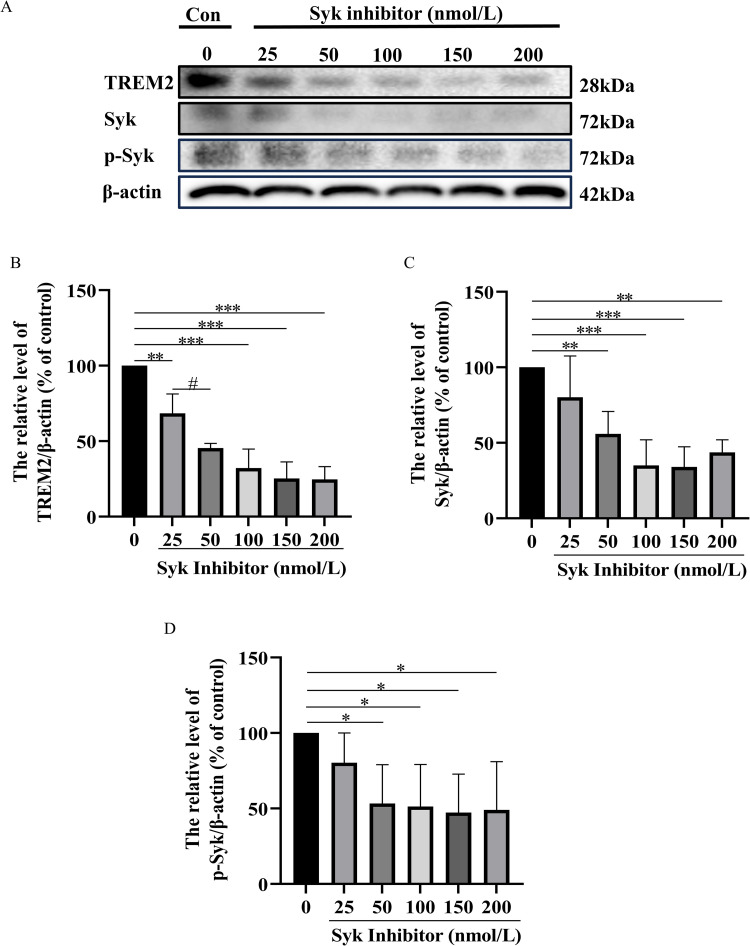
Effect of different concentrations of Syk inhibitor on the expressions of TREM2, Syk, and p-Syk HMC3 cells for 24 h. A, Western blot analysis showed the levels of TREM2, Syk, and p-Syk proteins in different concentrations of Syk inhibitor-treated HMC3 cells for 24 h. B, the bar graph shows the TREM2 protein expression level. C, the bar graph shows the Syk protein expression level. D, the bar graph shows the p-Syk protein expression level. Data represent the mean ± S.D. of three independent experiments, whereas * P < 0.05, ** P < 0.01, *** P < 0.001 versus control. # P < 0.05 versus the second group.

**Fig 8:**
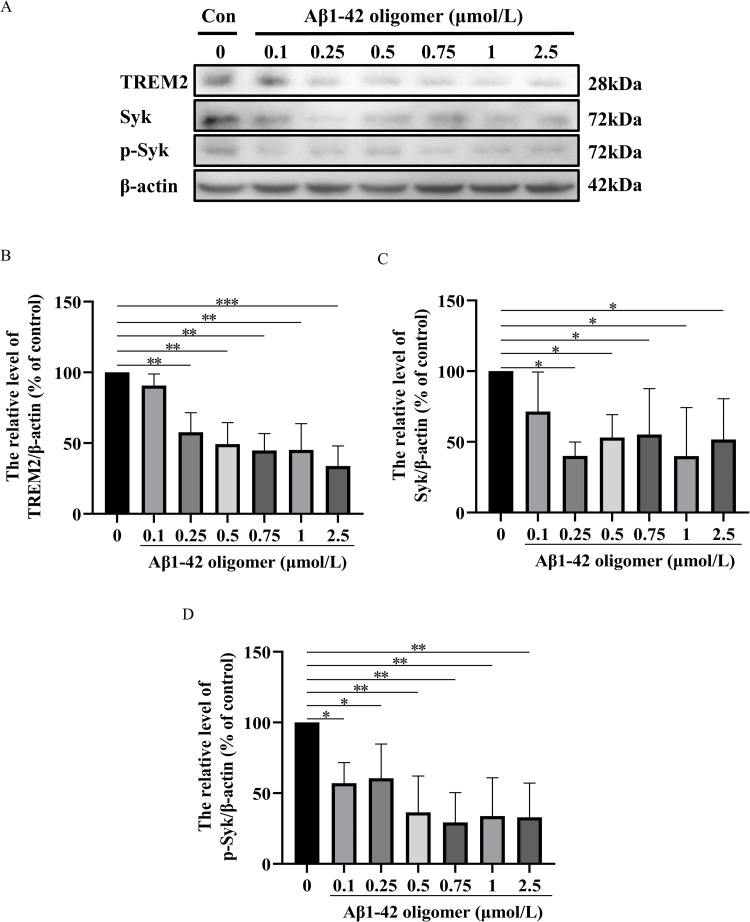
Effect of different concentrations of β-amyloid (1-42) oligomer on the expressions of TREM2, Syk, and p-Syk HMC3 cells for 24 h. A, Western blot analysis showed the levels of TREM2, Syk and p-Syk proteins in different concentrations of β-amyloid (1-42) oligomer-treated HMC3 cells for 24 h. B, the bar graph shows the TREM2 protein expression level. C, the bar graph shows the Syk protein expression level. D, the bar graph shows the p-Syk protein expression level. Data represent the mean ± S.D. of three independent experiments, whereas * P < 0.05, ** P < 0.01, *** P < 0.001 versus control.

To determine the appropriate concentrations of Syk inhibitor (SI) and Aβ oligomer (Aβ) for this experiment, HMC cells were respectively treated with different concentrations of SI (10, 25, 50, 100, 150, 200, 300, 400 and 500 nmol/L) and Aβ (0.1, 0.25, 0.5, 0.75, 1, 2.5, 5, 7.5 and 10 µmol/L) (S1 Fig) for 24 h. The cell viability was determined by the MTT assay. Based on the MTT assay, the result of Western blotting indicated that compared with the normal HMC3 cells, exposure of cells to SI-induced (50, 100, 150, and 200 nmol/L SI) or Aβ induced (0.25, 0.5, 0.75, 1, and 2.5 μmol/L Aβ) marked decrease in the ratio of TREM2, Syk and p-Syk (Figs 7 and 8). Our data showed that the respective treatment of 100 nmol/L SI and 2.5 µM Aβ for 24 h was the appropriate non-toxic concentration in cultured HMC3 cells.

Before exploring the protective impact of HAN, we detected the toxicity of HAN on HMC3 cells. HMC3 cells were incubated with different concentrations of HAN (0.1, 0.5, 1, 2.5, 5, 10, 25, 50, and 100 μmol/L) for 24 h. Then cell viability was determined by the MTT assay to detect the safety of HAN. There were no differences between HAN groups (< 100 μmol/L) and the control (S1 Fig). The results suggest that our experimental concentration gradient of HAN (0.5, 2.5, and 10 μmol/L) was safe for HMC3 cells.

### Regulation of HAN on Aβ 1–42 phagocytosis of HMC3 cells

**Fig 9:**
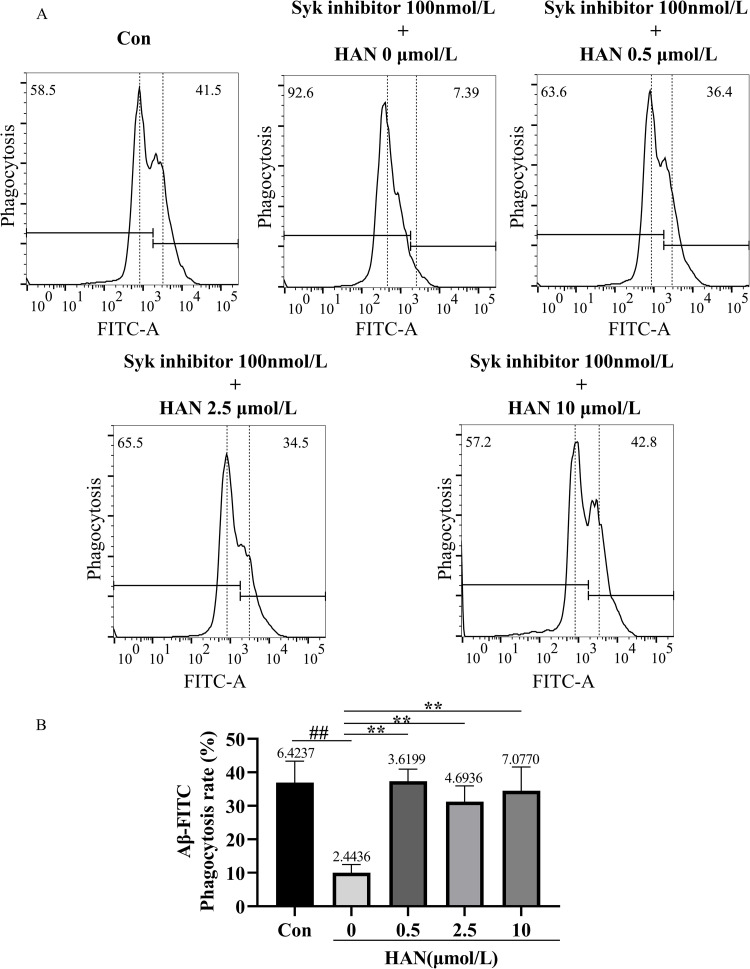
A, Effects of hancinone on phagocytic ability in 100 nM Syk inhibitor-induced HMC3 cells using 5-FITC-(Acp)- β-amyloid (1-42) staining assay. HMC3 cells were respectively treated with culture medium (control), 100 nM Syk inhibitor, 100 nM Syk inhibitor + 0.5 μM hancinone, 100 nM Syk inhibitor + 2.5 μM hancinone and 100 nM Syk inhibitor + 10 μM hancinone for 24 h. B, Histogram showed the percentage of cell phagocytosis rates after different treatments. The percentage of cell phagocytosis rates means the ratio of cell containing Aβ fluorescence to the total cells. ## P < 0.01 versus control. ** P < 0.01 versus the model group induced by 100 nM Syk inhibitor. Data represent the mean ± S.D. of three independent experiments.

**Fig 10:**
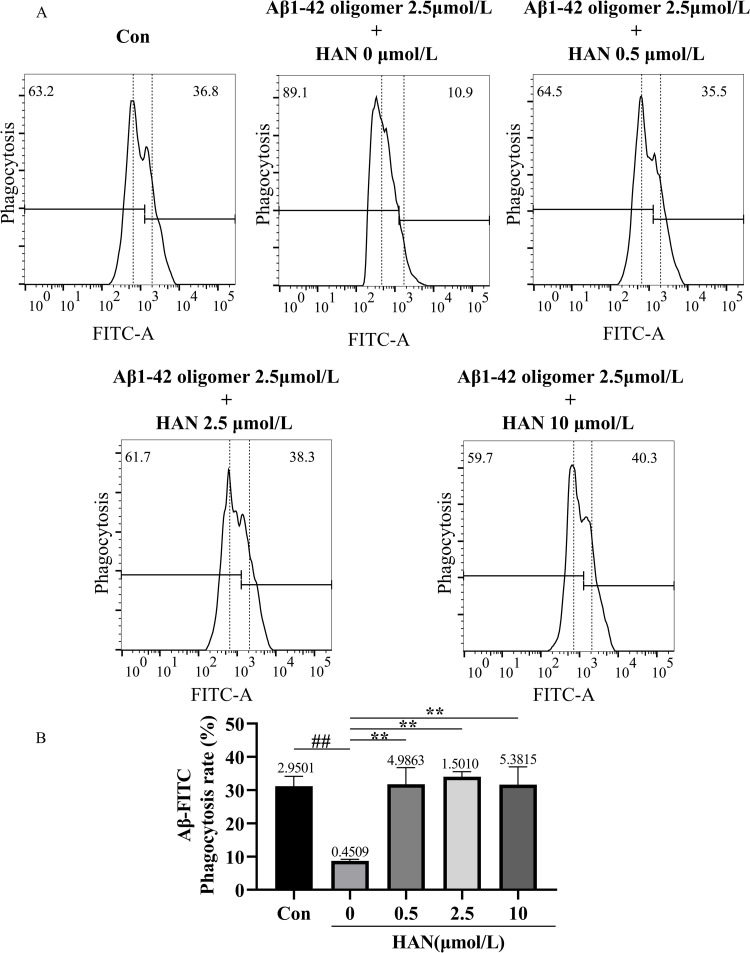
A, Effects of hancinone on phagocytic ability in 2.5 μmol/L Aβ1-42 oligomer-induced HMC3 cells using 5-FITC-(Acp)- β-amyloid (1-42) staining assay. HMC3 cells were respectively treated with culture medium (control), 2.5 μmol/L Aβ1-42 oligomer, 2.5 μmol/L Aβ1-42 oligomer + 0.5 μmol/L hancinone, 2.5 μmol/L Aβ1-42 oligomer + 2.5 μmol/L hancinone and 2.5 μmol/L Aβ1-42 oligomer + 10 μmol/L hancinone for 24 h. B, Histogram showed the percentage of cell phagocytosis rates after different treatments. ## P < 0.01 versus control. ** P < 0.01 versus the model group induced by 2.5 μmol/L Aβ1-42 oligomer. Data represent the mean ± S.D. of three independent experiments.

The phagocytic ability of HMC3 cells to Aβ oligomers was assessed using flow cytometric analysis. We reflect the amount of Aβ oligomers located within cells by observing the ratio of the number of positive cells after staining to the total number of cells detected. In the study of SI-induced HMC3 cells, the phagocytosis rates in the control (culture medium), model group (100 nmol/L SI), low-concentration HAN group (100 nmol/L SI + 0.5 μmol/L HAN), medium-concentration HAN group (100 nmol/L SI + 2.5 μmol/L HAN), and high-concentration HAN group (100 nmol/L SI + 10 μmol/L HAN) were 36.94%, 10.03%, 37.34%, 31.27% and 34.50% respectively (Fig 9A and 9B). In the study of Aβ-induced HMC3 cells, the phagocytosis rates in the control (culture medium), model group (2.5 μmol/L Aβ), low-concentration HAN group (2.5 μmol/L Aβ + 0.5 μmol/L HAN), medium-concentration HAN group (2.5 μmol/L Aβ + 2.5 μmol/L HAN) and high-concentration HAN group (2.5 μmol/L Aβ + 10 μmol/L HAN) were 30.99%, 8.81%, 31.45%, 33.55% and 31.55% respectively (Fig 10A and 10B). These results showed the significance of the difference in phagocytosis rates between the control and model groups in both two studies. Compared with the model group, the phagocytic ability of HMC3 cells to Aβ significantly increased in the low-concentration, medium-concentration, and high-concentration HAN groups (P < 0.01). Although there was no significant difference between the control, low-concentration HAN, medium-concentration HAN, and high-concentration HAN groups, the results indicated that suitable concentrations (0.5, 2.5 and 10 μmol/L) of HAN blocked the phagocytic injury to HMC3 cells caused by SI or Aβ (all P < 0.01; Figs 9B and 10B).

### HAN prevents SI -induced TREM2/Syk/PI3K/AKT/mTOR signaling pathway inhibition in HMC3 cells

**Fig 11:**
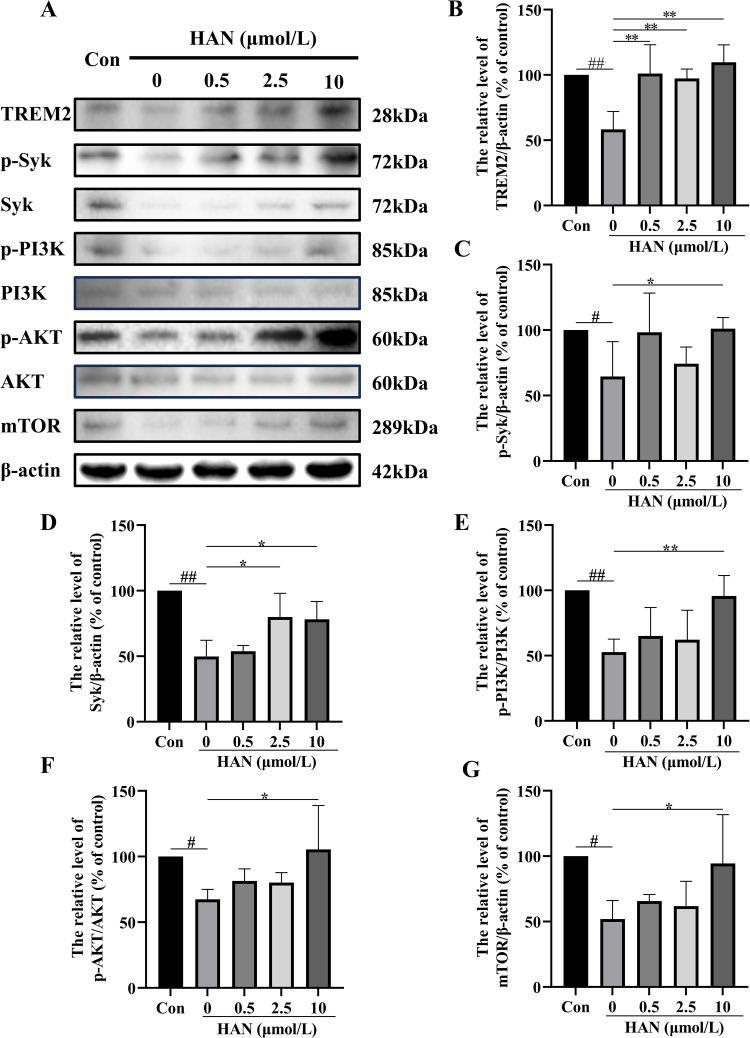
Effect of hancinone on the expressions of TREM2, Syk, p-Syk, p-PI3K, p-AKT, and mTOR in Syk inhibitor-induced HMC3 cells for 24 h. A, Western blot analysis showed the levels of TREM2, Syk, p-Syk, p-PI3K, p-AKT, PI3K, AKT, and mTOR proteins in Syk inhibitor-treated HMC3 cells for 24 h. B, the bar graph shows the TREM2 protein expression level. C, the bar graph shows the p-Syk protein expression level. D, the bar graph shows the Syk protein expression level. E, the bar graph shows the p-PI3K protein expression level. F, the bar graph shows the p-AKT protein expression level. G, the bar graph shows the mTOR protein expression level. Data represent the mean ± S.D. of three independent experiments, whereas # P < 0.05, ## P < 0.01 versus control. * P < 0.05, ** P < 0.01 versus the group treated with Syk inhibitor.

To further verify the effectiveness and mechanism of Syk inhibitor as an inhibitor of the TREM2/Syk signaling pathway, we examined phagocytosis-related protein expressions in HMC3 cells under SI. Compared with the normal HMC3 cells, exposure of cells to SI induced a marked decrease in the ratio of TREM2, Syk, p-Syk, p-PI3K, p-AKT, and mTOR. Treated with a high concentration of HAN in SI-stimulated cells, relative levels of TREM2, Syk, p-Syk, p-PI3K, p-AKT, and mTOR were significantly up-regulated (P < 0.05; Fig 11).

### HAN regulates the expression levels of TREM2/Syk/PI3K/AKT/mTOR signaling pathway-related proteins in Aβ-induced HMC3 cells

**Fig 12:**
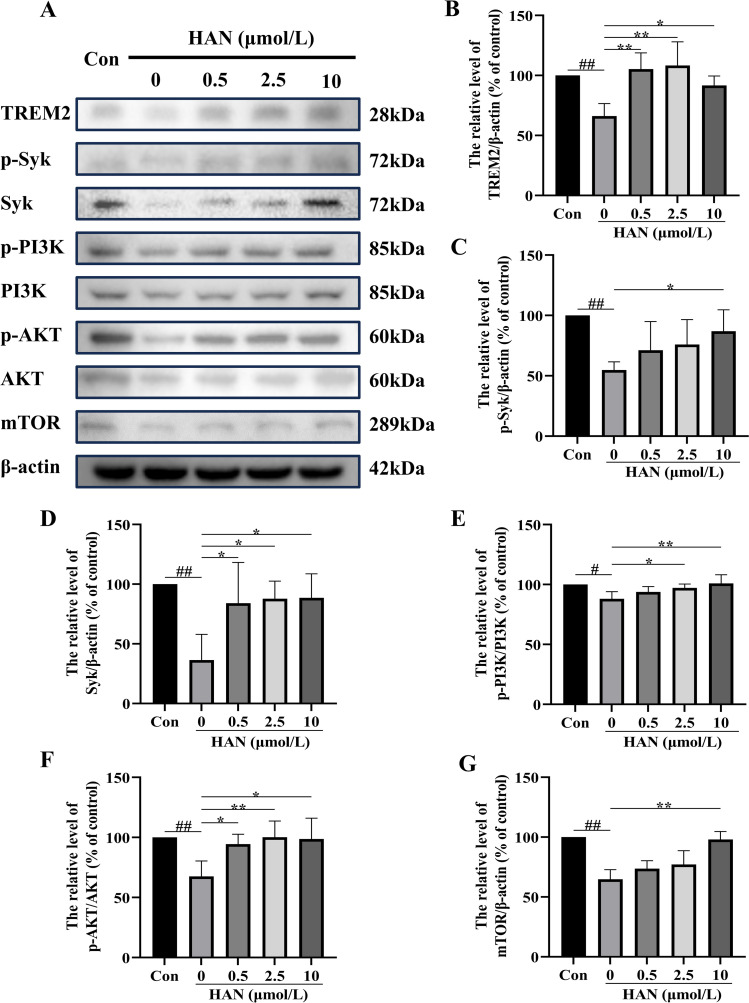
Effect of hancinone on the expressions of TREM2, Syk, p-Syk, p-PI3K, p-AKT, and mTOR in A β1-42 oligomer-induced HMC3 cells for 24 h. A, Western blot analysis showed the levels of TREM2, Syk, p-Syk, p-PI3K, p-AKT, PI3K, AKT, and mTOR proteins in Aβ1-42 oligomer-treated HMC3 cells for 24 h. B, the bar graph shows the TREM2 protein expression level. C, the bar graph shows the p-Syk protein expression level. D, the bar graph shows the Syk protein expression level. E, the bar graph shows the p-PI3K protein expression level. F, the bar graph shows the p-AKT protein expression level. G, the bar graph shows the mTOR protein expression level. Data represent the mean ± S.D. of three independent experiments, whereas # P < 0.05, ## P < 0.01 versus control. * P < 0.05, ** P < 0.01 versus the group treated with β-amyloid (1-42) oligomer.

Our flow cytometric analysis showed that the intervention of β-amyloid (1–42) oligomer could lead to defective Aβ phagocytosis in HMC3 cells (Fig 10B). To further investigate the influence of Aβ on HMC3 cells and to explore the mechanism by which HAN improves Aβ-induced phagocytic impairment, we detected TREM2, Syk, p-Syk, p-PI3K, PI3K, p-AKT, AKT and mTOR expression (Fig 12A). Compared with the control group, the expression levels of TREM2, Syk, p-Syk, p-PI3K, p-AKT and mTOR in the Model group (stimulated by Aβ) were significantly decreased (P < 0.05), while the expressions of TREM2, Syk, p-Syk, p-PI3K, p-AKT and mTOR were increased after treatment with high concentration of HAN (Fig 12B–12G). The results showed that the TREM2/Syk/PI3K/AKT/mTOR pathway played an important role in the Aβ-induced AD model, and HAN enhanced the function of the TREM2/Syk/PI3K/AKT/mTOR signaling pathway in Aβ-induced HMC3 cells.

### HAN facilitated the transition of HMC3 cells from the M1 phenotype to the M2 phenotype

**Fig 13:**
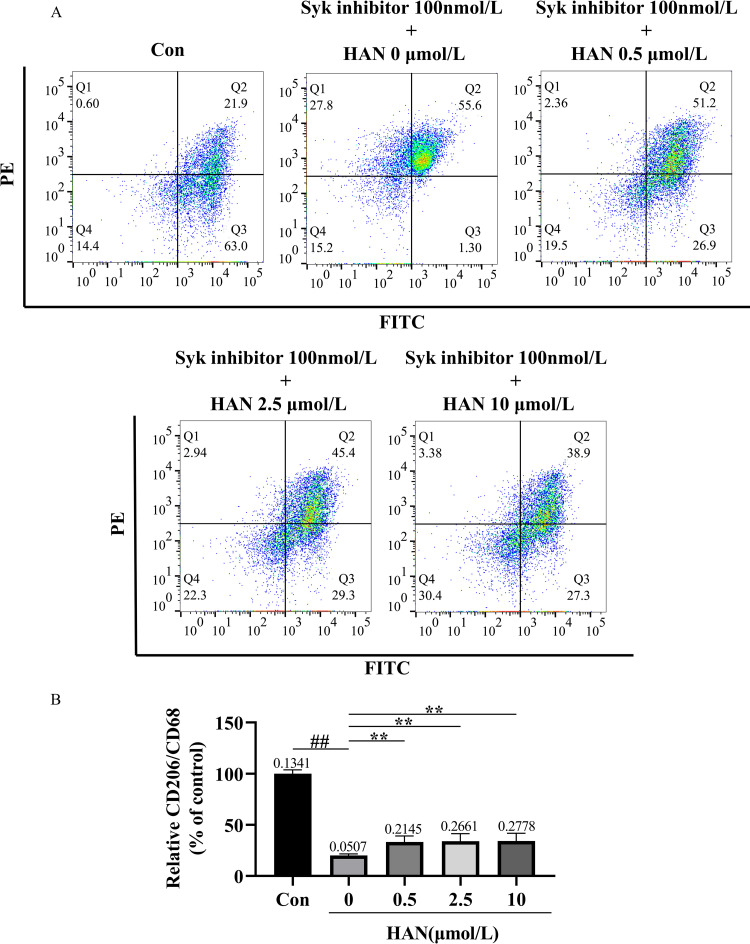
A, the transformation of phenotype in 100 nmol/L Syk inhibitor-induced HMC3 cells and effects of hancinone on phenotypic change using CD68 antibody and CD206 (MMR) antibody staining assay. HMC3 cells were respectively treated with culture medium (control), 100 nmol/L Syk inhibitor, 100 nmol/L Syk inhibitor + 0.5 μmol/L hancinone, 100 nmol/L Syk inhibitor + 2.5 μmol/L hancinone and 100 nmol/L Syk inhibitor + 10 μmol/L hancinone for 24 h. B, Histogram showed the percentage of CD206/CD68 after different treatments. ## P < 0.01 versus control. * P < 0.05 versus the model group induced by 100 nmol/L Syk inhibitor. Data represent the mean ± S.D. of three independent experiments.

**Fig 14:**
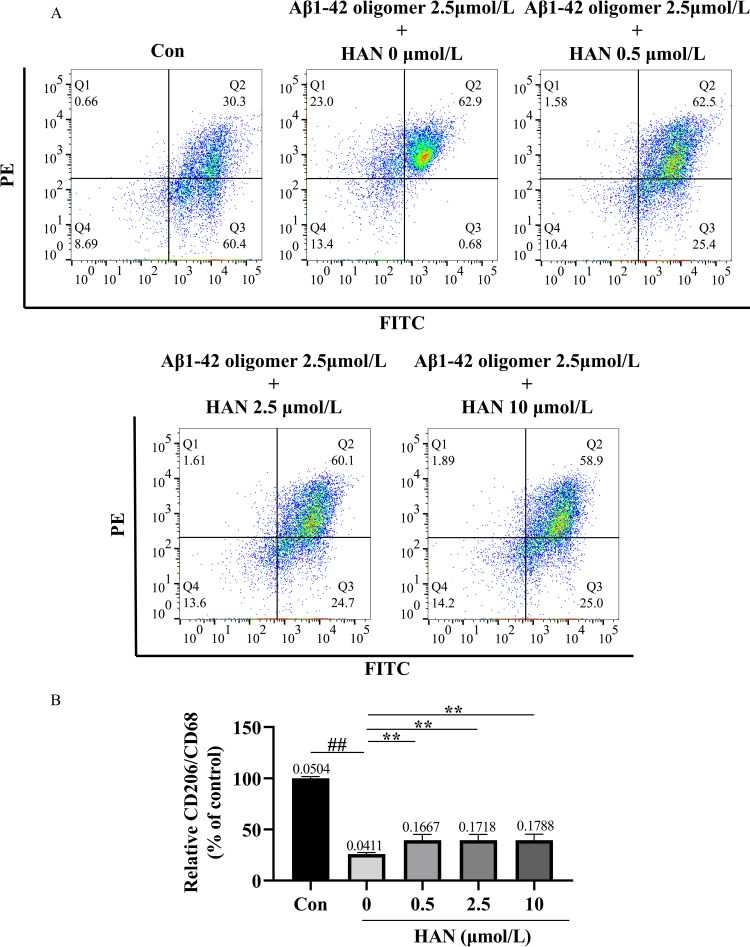
A, the transformation of phenotype in 2.5 μmol/L Aβ1-42 oligomer-induced HMC3 cells and effects of hancinone on phenotypic change using CD68 antibody and CD206 (MMR) antibody staining assay. HMC3 cells were respectively treated with culture medium (control), 2.5 μmol/L Aβ1-42 oligomer, 2.5 μmol/L Aβ1-42 oligomer + 0.5 μmol/L hancinone, 2.5 μmol/L Aβ1-42 oligomer + 2.5 μmol/L hancinone and 2.5 μmol/L Aβ1-42 oligomer + 10 μmol/L hancinone for 24 h. B, Histogram showed the percentage of CD206/CD68 after different treatments. ## P < 0.01 versus control. ** P < 0.01 versus the model group induced by 2.5 μmol/L Aβ1-42 oligomer. Data represent the mean ± S.D. of three independent experiments.

The transformation of HMC3 cells phenotype was detected by flow cytometric analysis (Figs 13A and 14A). In the research of SI-induced HMC3 cells, the relative CD206/CD68 in the control (culture medium), model group (100 nmol/L SI), low-concentration HAN group (100 nmol/L SI + 0.5 μmol/L HAN), medium-concentration HAN group (100 nmol/L SI + 2.5 μmol/L HAN), and high-concentration HAN group (100 nmol/L SI + 10 μmol/L HAN) were 100.00%, 25.71%, 38.76%, 39.59%, and 39.94%, respectively. In addition, in the research of Aβ-induced HMC3 cells, the relative CD206/CD68 in the control (culture medium), model group (2.5 μmol/L Aβ), low-concentration HAN group (2.5 μmol/L Aβ + 0.5 μmol/L HAN), medium-concentration HAN group (2.5 μmol/L Aβ + 2.5 μmol/L HAN) and high-concentration HAN group (2.5 μmol/L Aβ + 10 μmol/L HAN) were 100.00%, 25.98%, 39.53%, 39.41% and 39.42%, respectively. Compared with the control, the intervention of SI or Aβ in HMC3 cells significantly decreased the proportion of CD206/CD68 (P < 0.01; Figs 13B and 14B). Furthermore, the relative CD206/CD68 in the low-concentration, medium-concentration, and high-concentration HAN groups significantly increased compared with the model group (P < 0.05; Figs 13B and 14B). The result showed that SI or Aβ stimulated HMC3 cells and made them transform to the M1 phenotype, and HAN prevented this process by facilitating the transition of HMC3 cells from the M1 phenotype to the M2 phenotype.

## Discussion

In this study, we attempted to combine bioinformatics analysis, drug prediction, network pharmacology, molecular docking with laboratory experiments to screen and verify that hancinone, a component of PkO, has a therapeutic effect on AD. Hancinone may reduce the hazard of AD by binding to the core target Syk and regulating the TREM2/Syk/PI3K/AKT/mTOR signaling pathway. Consistent with the bioinformatics data, *in vitro* experiments indicated that hancinone could increase the TREM2, Syk, p-Syk and mTOR level compared with the model group. In addition, hancinone promotes the activation of HMC3 cells from the M1 phenotype to the M2 phenotype (Figs 13 and 14) and increases the content of Aβ oligomers in HMC3 cells. (Figs 9 and 10). Therefore, hancinone may have an impact on the phagocytic function of HMC3 cells. This systematic analysis provides new mechanisms concerning the therapeutic utility of hancinone for AD.

PkO is an herb frequently used to treat AD and exert neuroprotective effects in microglia [26]. Microglia are essential for Aβ clearance in AD as the sentinels of the brain. The variation of TREM2 and Syk affect the functional activity of microglia [6,7]. Moreover, functional downregulation of the TREM2, Syk, mTOR and decreased phosphorylation of PI3K/AKT occur in AD [27]. It is necessary to screen the ligands in PkO which up-regulate the TREM2, Syk or PI3K/AKT/mTOR signaling pathway. First, through the analysis of the PPI, GO, and KEGG of candidate targets, we found that numerous ligands in PkO may involve in the target Syk, mTOR and PI3K/AKT signaling pathway. Since Syk is one of the momentous nodes which represents the level of activation of neuroprotection in microglia and the downstream of TREM2 [27], we screened the monomers in PkO for Syk. After filtering, we detected that hancinone, kadsurenin K and kadsurenin M conjugated to Syk relatively efficiently. The previous study has showed that hancinone, an active ingredient of Piper hancei has the ability of inhibiting the production of nitric oxide secreted in lipopolysaccharide (LPS)-induced BV-2 microglial cells and play an anti-inflammatory role [28]. A study has shown that Syk coordinates neuroprotective microglial responses in neurodegenerative disease, and may increase the microglial phagocytosis of Aβ [29]. Pharmacoinformatics analysis, which combines computer-aided drug development with network pharmacology, can improve the efficiency of drug screening [30]. Thus, the conclusions that screen the active ingredients of Piper hancei to treat AD by this method are credible.

*In vitro* experiments, we found that the level of TREM2, p-PI3K, AKT, and mTOR significantly decreased under SI (Syk inhibitor, R406) or Aβ stimulation in HMC3 cells (Fig 11 and 12), and the phagocytic ability of SI- or Aβ-induced HMC3 cells to Aβ 1–42 was significantly impaired (Figs 9 and 10). Since the deficiency of TREM2 led to defect in PI3K/AKT/mTOR activation in microglia [7], TREM2 regulates downstream phosphorylation of Syk in microglia [27,31], Syk is the critical nodal point between the TREM2 and PI3K/AKT/mTOR signaling pathway in microglia. In addition, microglia is essential to the clearance of Aβ in AD [32]. TREM2 and Syk played critical roles in microglial phagocytosis of Aβ 1–42 [6,29]. Therefore, the phagocytosis defect of Aβ in Aβ-induced HMC3 cells is through impairing TREM2/Syk/PI3K/AKT/mTOR signaling pathway.

When SI- or Aβ-induced HMC3 cells were treat with HAN, the TREM2, Syk, p-Syk, p-PI3K, p-AKT, and mTOR expression all significantly upregulated in HMC3 cells treated by 10 μmol/L HAN (Figs 11 and 12). Compared with the model group, the phagocytic ability of HMC3 cells to Aβ significantly increased in HAN groups (0.5, 2.5 and 10 μmol/L) (Figs 9 and 10). The deposition of Aβ is the most important pathological feature and one of the critical causes of neurological damage in AD [33]. The previous study show that the absence of Syk in microglia leads to microglial impairment and PI3K/AKT signaling pathway downregulation, and pustulan-induced microglial CLEC7A activation can activate Syk and prevent the pathological process mentioned above [27]. The PI3K/AKT/mTOR signaling pathway upregulation perform a significant role in the nervous system [34]. The activation of Syk and TREM2 improved clearance of Aβ in microglia. The active P3IK/AKT/mTOR signaling pathway exhibited protection against Aβ-induced neurotoxicity [29,35]. Thus hancinone may promote the ability of HMC3 Cells to phagocytosis of Aβ through regulating TREM2/Syk/PI3K/AKT/mTOR signaling pathway.

Interestingly, previous research suggested that M2-phenotype microglia exhibited better phagocytosis of Aβ than M1-phenotype, alleviated excessive inflammatory responses, and ameliorated cognitive impairment in AD mice [36]. When attacked by foreign pathogens or toxins, microglia polarize into the M1 phenotype, with the increasing of main phenotypic marker CD68, release pro-inflammatory factors such as IL-6 and tumor necrosis factor-alpha (TNF-α), and produce a neuroinflammatory response. When the cellular environment changes, microglia can polarize from an M1-activated phenotype to M2-activated phenotype. The main marker for the M2 phenotype of microglia is CD206. The M2 microglia mainly secrete anti-inflammatory factors such as IL-4 and IL-10 [37,38] and inhibit the neuroinflammatory response induced by Aβ oligomerization [39]. We used a Flow cytometry assay in which HMC3 cells were orderly stained by CD68 antibody and CD206 (MMR) antibody to analyze the transformation of phenotype. The results of Flow cytometry indicated that SI or Aβ stimulated HMC3 cells and made them transform to M1 phenotype (Figs 13 and 14). Therefore, the extracellular accumulation of Aβ in HMC3 cells could lead to the phenotypic change from M2 to M1 probably due to the defect of Syk. Our results showed that HAN could prevent this pathological process and promote the activation of HMC3 cells from the M1 phenotype to the M2 phenotype (Figs 13 and 14), which might impact ability of HMC3 Cells to phagocytosis of Aβ. The low expression level of TREM2 in HMC3 cells decrease ability to phagocytose Aβ. Also, SI or Aβ stimulation HMC3 cells affect the expression of CD68 and CD206. We speculated that SI might possess a similar effect to Aβ on microglia phagocytosis. Previous studies showed that Syk signaling could differentially modulate M1-like and M2-like macrophage phenotype and function. The change of the expression of TREM2 could affect the M1/M2 polarization of microglia in AD model [40,41]. Combining previous studies and our experimental results, we deduce that HAN promote the activation of HMC3 cells from the M1 phenotype to the M2 phenotype probably through regulating TREM2 and Syk signal.

This study has limitations that need to be acknowledged. We screened the active ingredients from our database, but it is possible that we may have missed some ligands outside the range of the database. We did not experimentally validate the effect of the other two monomers which bind well to the receptors. MTT is an indirect measurement method, which may be influenced by cell type, status and MTT toxicity. Although our study has confirmed changes in protein expression along the pathway. To demonstrate the more complete role of this pathway in cellular phagocytosis, knock-out or overexpression studies of specific proteins are necessary. Further refinement and exploration are required in the future research. In our study on the phagocytic function of microglia, we explored it from two aspects: staining and phenotypic changes. Next, the phagocytic process under the microscope and using more specific markers are used. Additionally, our findings were validated in an SI- or Aβ-induced AD cell model. The experiments *in vivo* will be included in the future studies.

## Conclusions

In this study, we used bioinformatics analysis, drug forecasting, network pharmacology, and molecular docking to improve the efficiency of experimental validation of the active ingredients of herbal medicines. Ultimately, hancinone was recognized as a potential therapeutic candidate for AD. Hancinone facilitated the transition of HMC3 cells from the M1 phenotype to the M2 phenotype, enhanced the phagocytic capability of HMC3 cells to Aβ, and reduced neurotoxicity through a certain degree of influence on TREM2/Syk/PI3K/AKT/mTOR signaling pathway.

## Supporting information

S1 Fig Cell viability and drug safety assay. A, HMC3 cells were incubated with increasing concentrations of Syk inhibitor (10, 25, 50, 100, 150, 200, 300, 400 and 500 nmol/L) for 24 h. B, HMC3 cells were incubated with increasing concentrations of Aβ1–42 (0.1, 0.25, 0.5, 0.75, 1, 2.5, 5, 7.5 and 10 µmol/L) for 24 h. C, HMC3 cells were incubated with increasing concentrations of hancinone (0.1, 0.5, 1, 2.5, 5, 10, 25, 50 and 100 µmol/L) for 24 h. Cell viability was assessed using the MTT assay. * P < 0.05, ** P < 0.01 significantly compared with control group.
(DOCX)

S2 Fig Transmission Electron Microscope (TEM) of Aβ1–42 oligomer. Negative stain transmission electron microscopy images acquired at 80 kV. Scale bar = 500 nm.
(DOCX)

S3 Fig The chemical structure of hancinone. (DOCX)

S1 Table The 13 active ingredients from *Piper kadsura* and related ADME values. (DOCX)

S2 Table The compounds of PkO we used in the study from TCMSP database. (DOCX)

S3 Table The information of antibodies and reagents we used in the study. (DOCX)

S1 Report The HPLC and MS analysis of β-Amyloid (1–42). (PDF)

S1 File Raw_images. (PDF)
