# Supplementary material for: Hancinone possesses potentials on increasing the ability of HMC3 cells to phagocytosis of Aβ1-42 via TREM2/Syk/PI3K/AKT/mTOR signaling pathway
Source: PLoS One. 2025 May 27;20(5):e0324202. doi: 10.1371/journal.pone.0324202 (PMC12111670; doi:10.1371/journal.pone.0324202)

**S1 Fig.** Cell viability and drug safety assay. **A,** HMC3 cells were incubated with increasing concentrations of Syk inhibitor (10, 25, 50, 100, 150, 200, 300, 400 and 500 nmol/L) for 24 h. **B,** HMC3 cells were incubated with increasing concentrations of Aβ1-42 (0.1, 0.25, 0.5, 0.75, 1, 2.5, 5, 7.5 and 10 µmol/L) for 24 h. **C,** HMC3 cells were incubated with increasing concentrations of hancinone (0.1, 0.5, 1, 2.5, 5, 10, 25, 50 and 100 µmol/L) for 24 h. Cell viability was assessed using the MTT assay. * P < 0.05, ** P < 0.01 significantly compared with control group.


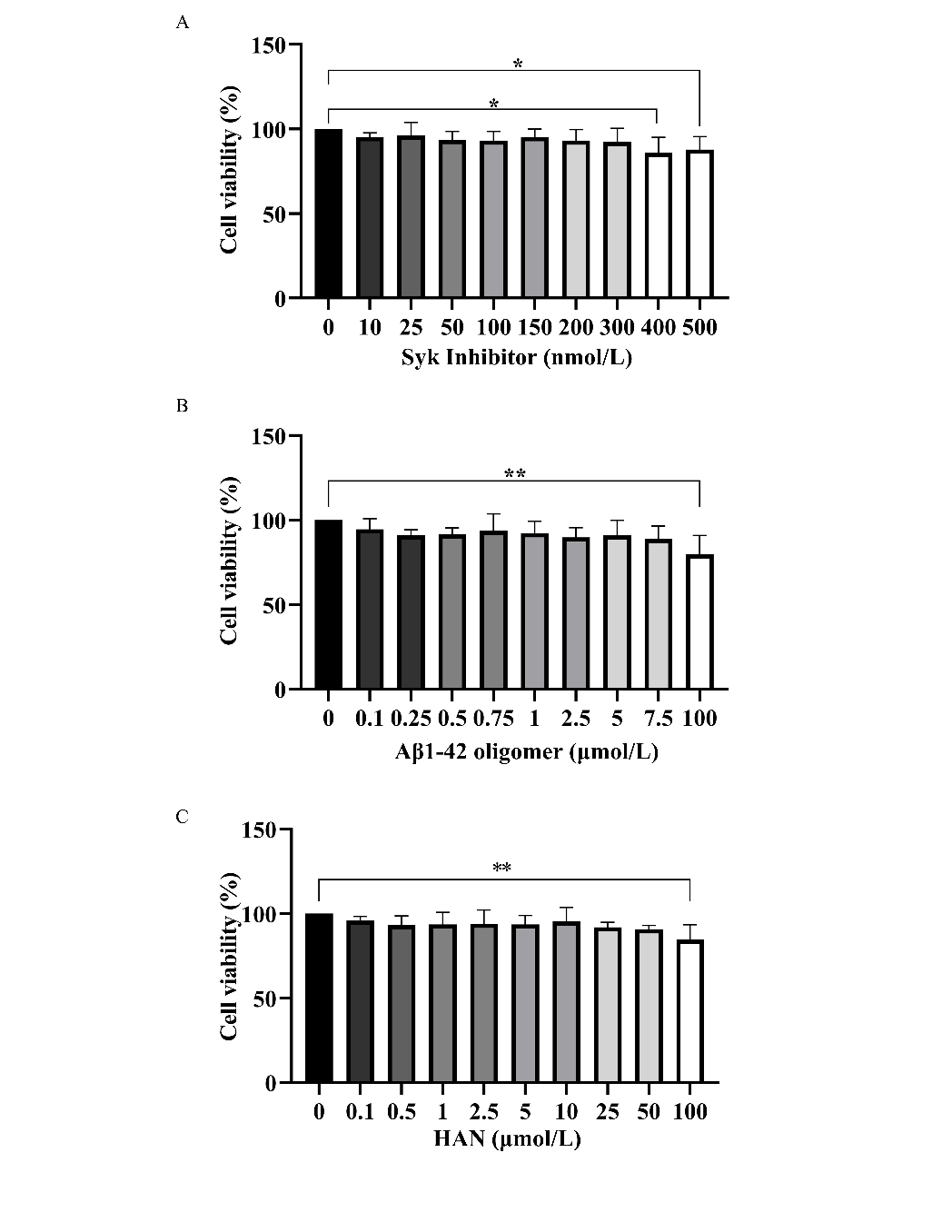

Supplement: S1 Fig — A, HMC3 cells were incubated with increasing concentrations of Syk inhibitor (10, 25, 50, 100, 150, 200, 300, 400 and 500 nmol/L) for 24 h. B, HMC3 cells were incubated with increasing concentrations of Aβ1–42 (0.1, 0.25, 0.5, 0.75, 1, 2.5, 5, 7.5 and 10 µmol/L) for 24 h. C, HMC3 cells were incubated with increasing concentrations of hancinone (0.1, 0.5, 1, 2.5, 5, 10, 25, 50 and 100 µmol/L) for 24 h. Cell viability was assessed using the MTT assay. * P < 0.05, ** P < 0.01 significantly compared with control group. (DOCX) [file pone.0324202.s001.docx]
