# Supplementary material for: Hancinone possesses potentials on increasing the ability of HMC3 cells to phagocytosis of Aβ1-42 via TREM2/Syk/PI3K/AKT/mTOR signaling pathway
Source: PLoS One. 2025 May 27;20(5):e0324202. doi: 10.1371/journal.pone.0324202 (PMC12111670; doi:10.1371/journal.pone.0324202)

**S2 Fig.** Transmission Electron Microscope (TEM) of Aβ1-42 oligomer. Negative stain transmission electron microscopy images acquired at 80 kV. Scale bar = 500 nm.


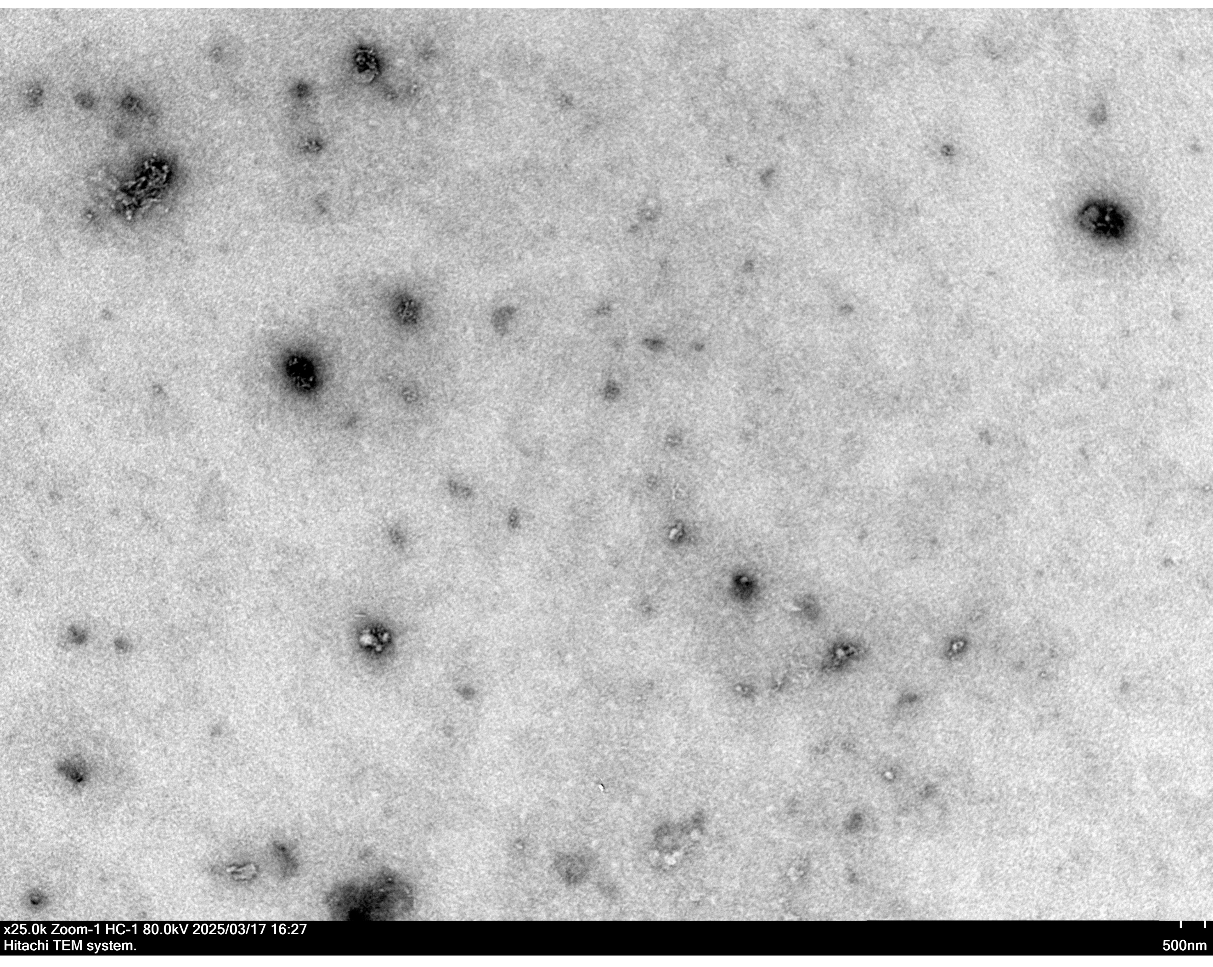

Supplement: S2 Fig — Negative stain transmission electron microscopy images acquired at 80 kV. Scale bar = 500 nm. (DOCX) [file pone.0324202.s002.docx]
