# Supplementary material for: Hancinone possesses potentials on increasing the ability of HMC3 cells to phagocytosis of Aβ1-42 via TREM2/Syk/PI3K/AKT/mTOR signaling pathway
Source: PLoS One. 2025 May 27;20(5):e0324202. doi: 10.1371/journal.pone.0324202 (PMC12111670; doi:10.1371/journal.pone.0324202)
Supplement: S1 Table — (DOCX) [file pone.0324202.s004.docx]

**S1 Table. The 13 active ingredients from Piper kadsura and ADME values.**

| **Number** | **Molecule name** | **Lipinski Rules** | | | | **Lipinski’s Violations** | **Bioavailability Score** | **TPSA (Å2)** | **GI absorption** | **BBB permeant** |  |
| --- | --- | --- | --- | --- | --- | --- | --- | --- | --- | --- | --- |
|  |  |  |  |  |  |  |  |  |  |  |  |
|  |  | **MW** | **HBA** | **HBD** | **Mlog p** |  |  |  |  |  |  |
|  |  | **<500** | **<10** | **≤5** | **≤4.15** | **≤1** | **>0.1** | **<90** |  |  |  |
| 1 | (2R,3R,3aS)-3a-allyl-2-(1,3-benzodioxol-5-yl)-5  -methoxy-3-methyl-2,3-dihydrobenzofuran-6-one | 340.37 | 5 | 0 | 1.77 | 0 | 0.85 | 53.99 | High | Yes |  |
| 2 | Denudatin B | 356.41 | 5 | 0 | 1.59 | 0 | 0.85 | 53.99 | High | Yes |  |
| 3 | futokadsurin C | 356.41 | 5 | 0 | 2.61 | 0 | 0.55 | 46.15 | High | Yes |  |
| 4 | Galgravin | 372.45 | 5 | 0 | 2.42 | 0 | 0.55 | 46.15 | High | Yes |  |
| 5 | (2S,3S,4S,5S)-2,5-bis(3,4-dimethoxyphenyl)  -3,4-dimethyltetrahydrofuran | 372.45 | 5 | 0 | 2.42 | 0 | 0.55 | 46.15 | High | Yes |  |
| 6 | hancinone | 340.37 | 5 | 0 | 1.77 | 0 | 0.85 | 53.99 | High | Yes |  |
| 7 | Bicyclo(3.2.1)oct-3-ene-2,8-dione,7  -(4-hydroxy-3-methoxyphenyl)-5-methoxy  -6-methyl-3-(2-propenyl)-,(1R-(6-endo,7-exo))- | 342.39 | 5 | 1 | 1.36 | 0 | 0.55 | 72.83 | High | Yes |  |
| 8 | (2S,3S)-2-(3,4-dimethoxyphenyl)-7-methoxy-3-methyl  -2,3-dihydrobenzofuran-5-carbaldehyde | 328.36 | 5 | 0 | 1.67 | 0 | 0.55 | 53.99 | High | Yes |  |
| 9 | Kadsurenone | 356.41 | 5 | 0 | 1.59 | 0 | 0.85 | 53.99 | High | Yes |  |
| 10 | Kadsurin A | 372.41 | 6 | 0 | 1.66 | 0 | 0.55 | 63.22 | High | Yes |  |
| 11 | (4R)-2-allyl-4-[(E)-2-(4-hydroxy-3-methoxyphenyl)-1  -methylvinyl]-4,5-dimethoxy-1-cyclohexa-2,5-dienone | 356.41 | 5 | 1 | 1.51 | 0 | 0.85 | 64.99 | High | Yes |  |
| 12 | wallichinine | 370.44 | 5 | 0 | 1.73 | 0 | 0.85 | 53.99 | High | Yes |  |
| 13 | futoquinol | 354.4 | 5 | 0 | 1.91 | 0 | 0.85 | 53.99 | High | Yes |  |
