# Supplementary material for: Hancinone possesses potentials on increasing the ability of HMC3 cells to phagocytosis of Aβ1-42 via TREM2/Syk/PI3K/AKT/mTOR signaling pathway
Source: PLoS One. 2025 May 27;20(5):e0324202. doi: 10.1371/journal.pone.0324202 (PMC12111670; doi:10.1371/journal.pone.0324202)
Supplement: S2 Table — (DOCX) [file pone.0324202.s005.docx]

**S2 Table. The compounds of PkO from TCMSP database**

| Number | Molecule name |
| --- | --- |
| 1  2  3  4  5  6  7  8  9  10  11  12  13  14  15  16  17  18  19  20  21  22  23  24  25  26  27  28  29  30  31 | (+)-Crotepoxide  aristololactam A IIIa  (2R,3R,3aS)-3a-allyl-2-(1,3-benzodioxol-5-yl)-5-methoxy-3-methyl-2,3-dihydrobenzofuran-6-one  denudatin,a  Denudatin B  futoenone  futokadsurin C  Galgravin  (2S,3S,4S,5S)-2,5-bis(3,4-dimethoxyphenyl)-3,4-dimethyltetrahydrofuran  hancinone  isofutoquinol A  Kadsurenin B  (1R,5S,6R,7R,8R)-3-allyl-6-(3,4-dimethoxyphenyl)-8-hydroxy-1-methoxy-7-methyl-4-bicyclo[3.2.1]oct-2-enone  Bicyclo(3.2.1)oct-3-ene-2,8-dione, 7-(4-hydroxy-3-methoxyphenyl)-5-methoxy-6-methyl-3-(2-propenyl)-, (1R-(6-endo,7-exo))-  acetic acid [(1R,5S,6R,7R,8R)-3-allyl-6-(3,4-dimethoxyphenyl)-1-methoxy-7-methyl-4-oxo-8-bicyclo[3.2.1]oct-2-enyl] ester  (2S,3S)-2-(3,4-dimethoxyphenyl)-7-methoxy-3-methyl-2,3-dihydrobenzofuran-5-carbaldehyde  Kadsurenone  Kadsurin A  kadsurin B  (2R,3R)-2-(3,4-dimethoxyphenyl)-7-methoxy-3-methyl-5-[(E)-prop-1-enyl]-2,3-dihydrobenzofuran  liliflone  (4R)-2-allyl-4-[(E)-2-(4-hydroxy-3-methoxyphenyl)-1-methylvinyl]-4,5-dimethoxy-1-cyclohexa-2,5-dienone  piperkadsin B  piperkadsin C  piperlactam S  Stigmasterol  n-coumaroyltyramine  Veraguensin  wallichinine  futoamide  futoquinol |
