# Supplementary material for: Hancinone possesses potentials on increasing the ability of HMC3 cells to phagocytosis of Aβ1-42 via TREM2/Syk/PI3K/AKT/mTOR signaling pathway
Source: PLoS One. 2025 May 27;20(5):e0324202. doi: 10.1371/journal.pone.0324202 (PMC12111670; doi:10.1371/journal.pone.0324202)
Supplement: S3 Table — (DOCX) [file pone.0324202.s006.docx]

**S3 Table. Antibodies and reagents**

| Antibody | Species and Source | Catalogue Number | Dilution | Supplier |
| --- | --- | --- | --- | --- |
| Phospho-Syk | Rabbit mAb | #2710 | 1:1000 | Cell Signaling Technology (Denver, USA) |
| Syk | Rabbit mAb | #13198 | 1:1000 | Cell Signaling Technology (Denver, USA) |
| Phospho- AKT | Rabbit mAb | #4060 | 1:2000 | Cell Signaling Technology (Denver, USA) |
| Pan-AKT | Rabbit mAb | A18675 | 1:1000 | ABclonal Biotech Co., Ltd. (Wuhan, CHINA) |
| phospho-PI3K | Rabbit pAb | AP0427 | 1:1000 | ABclonal Biotech Co., Ltd. (Wuhan, CHINA) |
| PI3 Kinase  β-actin  HRP Goat Anti-Rabbit IgG (H+L)  TREM2  mTOR  CD68  CD206  β-Amyloid (1-42)  MTT  DMSO  FBS  SDS-PAGE  Primary Antibody Dilution Buffer  Secondary Antibody Dilution Buffer  Trypsin Solution without EDTA | Rabbit mAb  Rabbit mAb  Goat anti-Rabbit  Rabbit pAb  Rabbit mAb  Mouse mAb  Mouse mAb  -  -  -  Fetal bovine  -  -  -  - | A4992  AC026  AS014  AF09731  ab134903  12-0689-42  141703  052487  M2128  472301  A5256701  P0015A  P0023A-500ml  P0023D-500ml  C0205 | 1:1000  1:50000  1:2000  1:500  1:10000  1:50  1:2000  -  -  -  -  1X  -  -  - | ABclonal Biotech Co., Ltd. (Wuhan, CHINA)  ABclonal Biotech Co., Ltd. (Wuhan, CHINA)  ABclonal Biotech Co., Ltd. (Wuhan, CHINA)  AiFang Biological Co., Ltd. (Hunan, China)  Abcam (Cambridge, England)  Invitrogen (ThermoFisher Scientific, USA)  Biolegend (CA, USA)  GL Biochem Co., Ltd. (Shanghai, China)  Sigma-Aldrich (St. Louis, USA)  Sigma-Aldrich (St. Louis, USA)  GIBCO (Australia)  Beyotime (Shanghai, China)  Beyotime (Shanghai, China)  Beyotime (Shanghai, China)  Beyotime (Shanghai, China) |
