# Supplementary material for: Hancinone possesses potentials on increasing the ability of HMC3 cells to phagocytosis of Aβ1-42 via TREM2/Syk/PI3K/AKT/mTOR signaling pathway
Source: PLoS One. 2025 May 27;20(5):e0324202. doi: 10.1371/journal.pone.0324202 (PMC12111670; doi:10.1371/journal.pone.0324202)

The HPLC and MS Analysis of β-Amyloid(1-42)

Product Name: β-Amyloid(1- 42),human

Purity: 96.30%

Molecular Weight: 4514.14

Solubility: 1mg/ml in 50%ACN/50%H_2O

Test	Specification	Result
**Purity:**	HPLC	Conforms
	(See attached RP-HPLC chromatogram)	
**MS Analysis:**	ESI-MS	Conforms
	(See attached MS spectrum)	
**Counter Ion:**	Trifluoroacetate	Conforms
**Appearance:**	Lyophilized powder or Crystallization	Conforms

Important: The peptides can be used for research only. Most of the peptides are lyophilized white or faint yellow powder while fluorescent modified ones have special colors. The state of peptides with strong hydrophilic properties may be crystalline or liquid which does not affect for use. Before experiment, please choose proper solvent for your experiment to dissolve peptides. If peptides cannot be dissolved under harsh conditions, we can carry out feasibility study. Storage conditions:-20ºC, seal, avoid light, dry.

Please test the sample within two weeks after receiving it.

HPLC Analysis Report

Measurement:	Peak Area	Run Time:	20min
Calculation Type:	Percent	Wavelength :	220nm
Flow Rate :	1.0ml/min	Inj.Vol:	10uL
Column:	Kromasil 100-5C18,4.6mmX250mm,5 micron	Column Temp:	25℃
Buffer A :	0.1%TFA in Acetonitrile	Buffer B:	0.1%TFA in water

Gradient(linear):		A	B
	0.0min	30%	70%
	20min	80%	20%
	20.1min	100%	0%

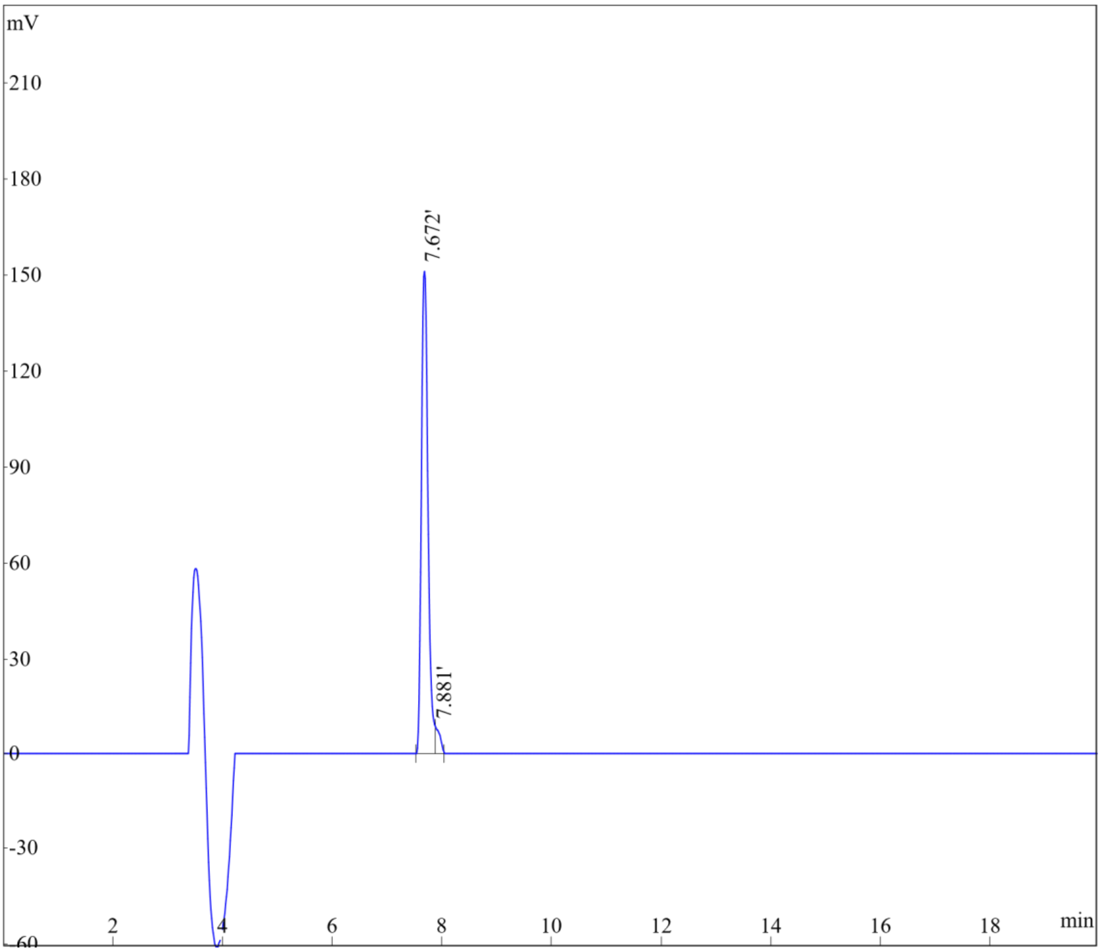

MS Analysis Report

Ion Source: ESI | Capillary(V):±(2500~3500)
Desolvation(L/hr):800 | Desolvation Temp:450℃
Cone(V): 15~30 | Run Time: 1min

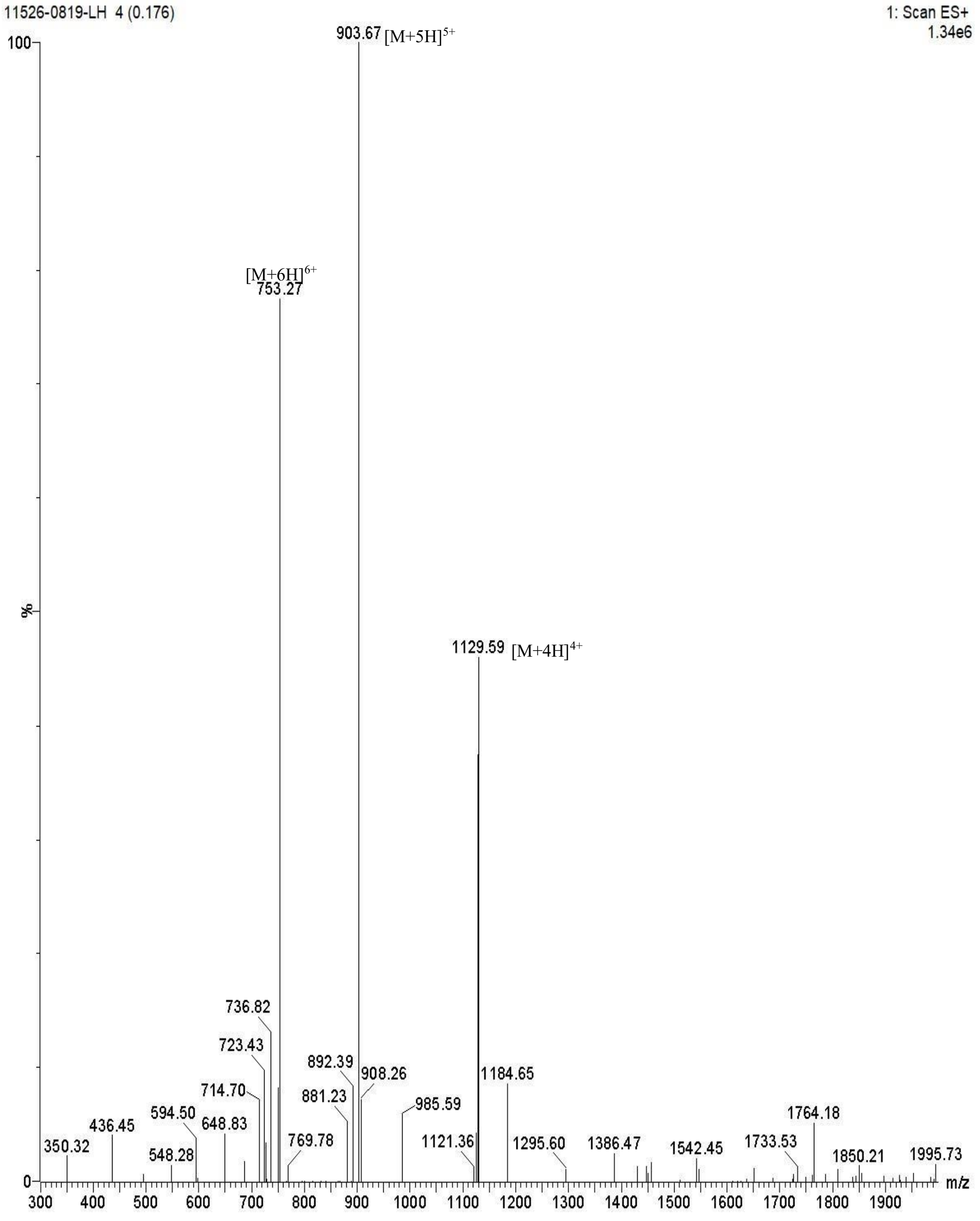

Supplement: S1 Report — (PDF) [file pone.0324202.s007.pdf]
