# Supplementary material for: Hancinone possesses potentials on increasing the ability of HMC3 cells to phagocytosis of Aβ1-42 via TREM2/Syk/PI3K/AKT/mTOR signaling pathway
Source: PLoS One. 2025 May 27;20(5):e0324202. doi: 10.1371/journal.pone.0324202 (PMC12111670; doi:10.1371/journal.pone.0324202)
Supplement: S1 File — (PDF) [file pone.0324202.s008.pdf]

Fig 7

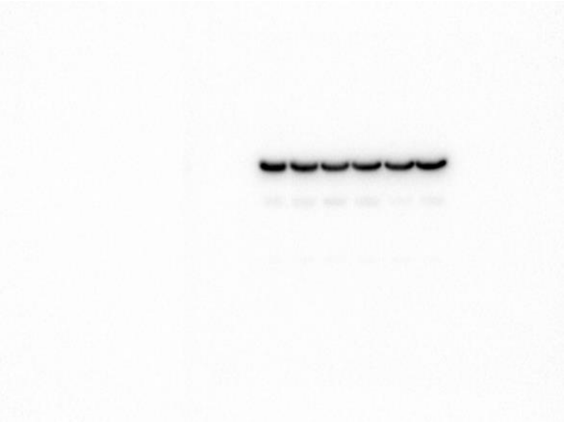

β-actin

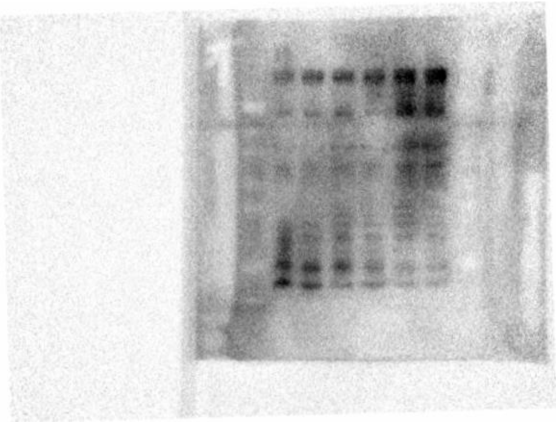

p-Syk

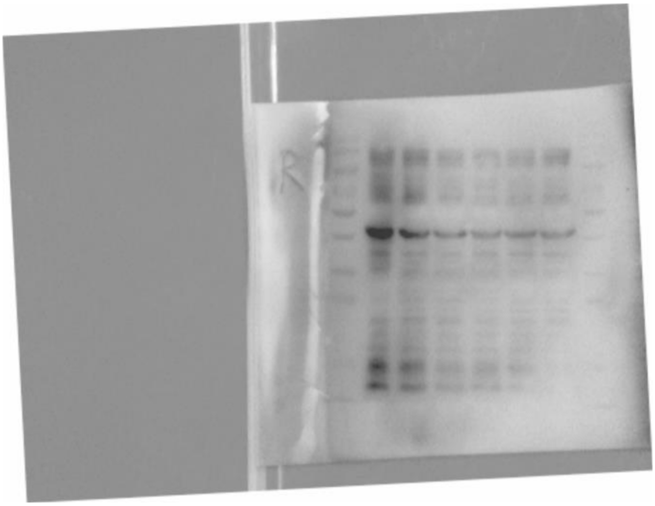

Syk

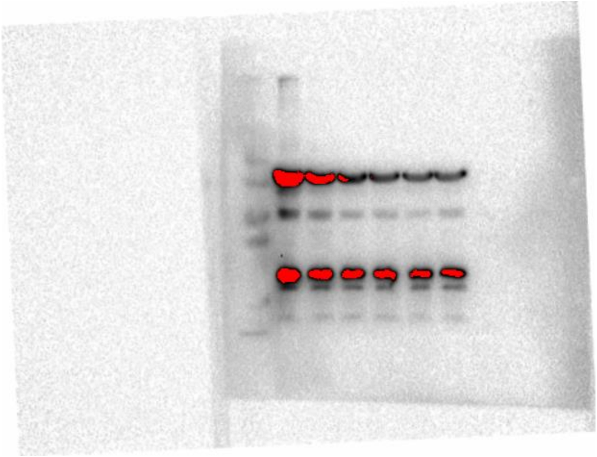

TREM-2

Fig 8

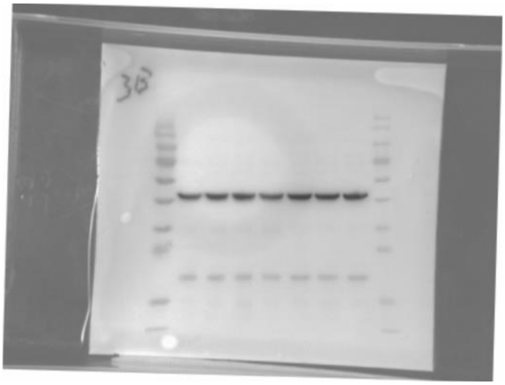

β-actin

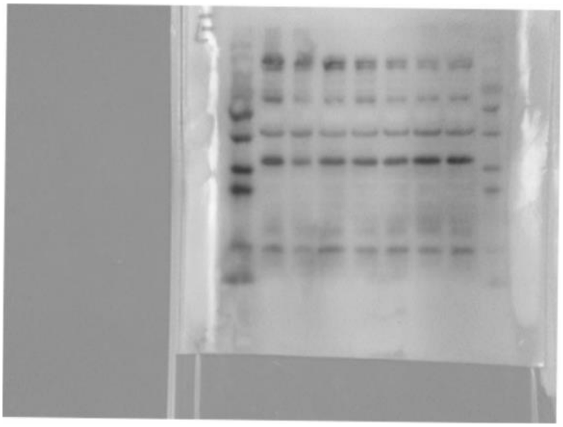

p-Syk

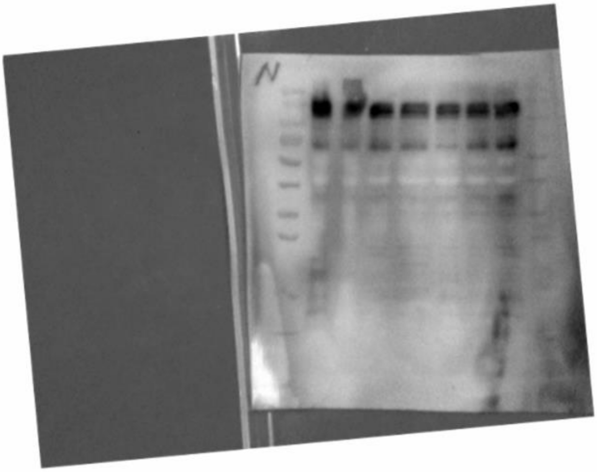

Syk

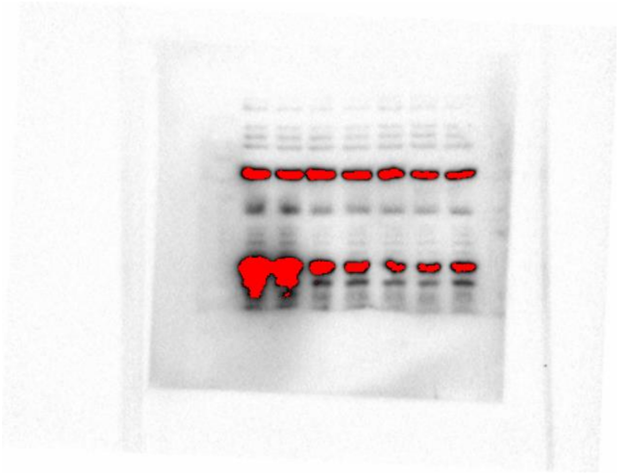

TREM-2

Fig 11

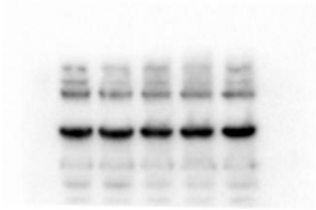

β-actin

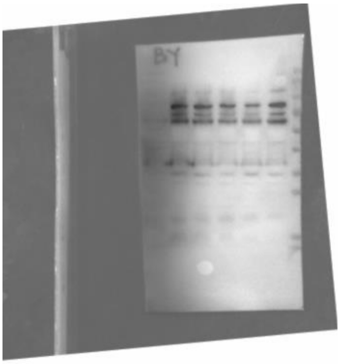

AKT

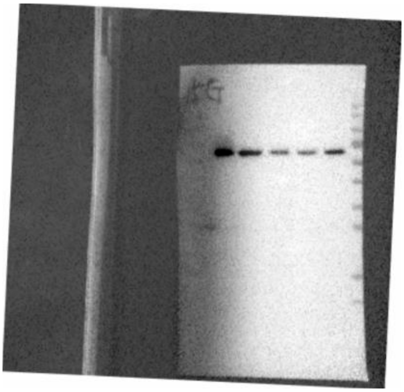

P-AKT

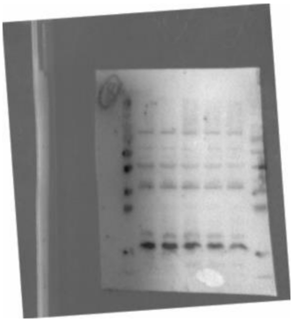

PI3K

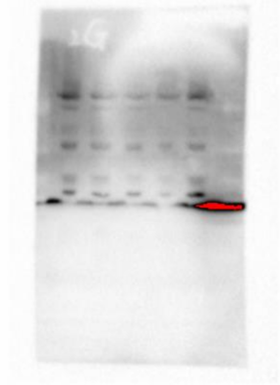

p-PI3K

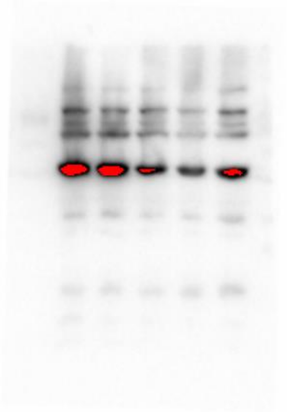

p-Syk

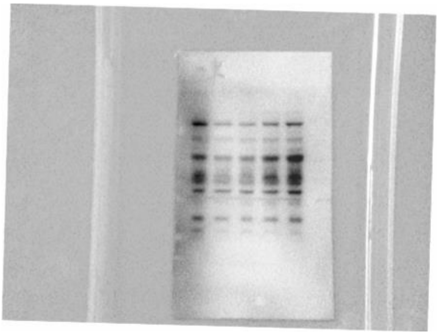

Syk

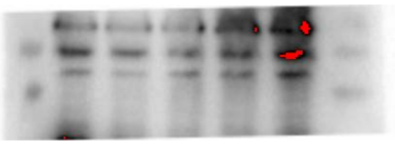

TREM2

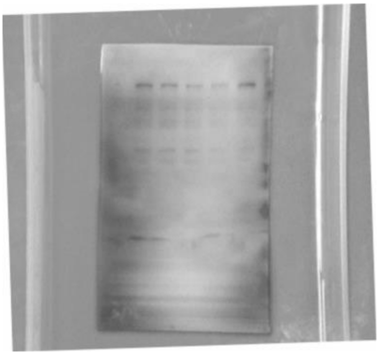

mTOR

Fig 12

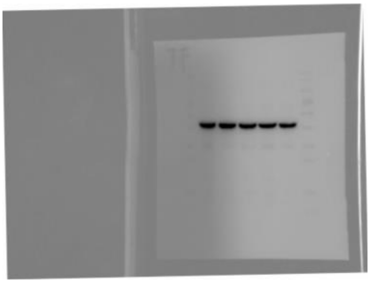

β-actin

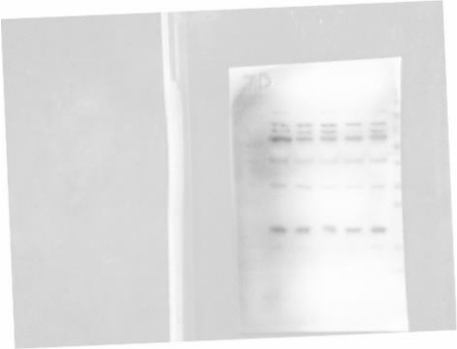

AKT

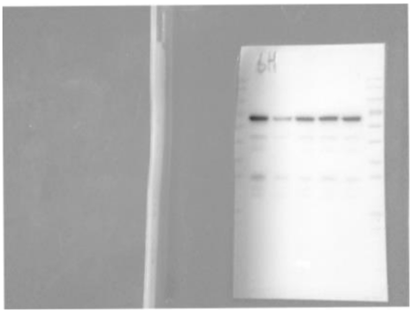

P-AKT

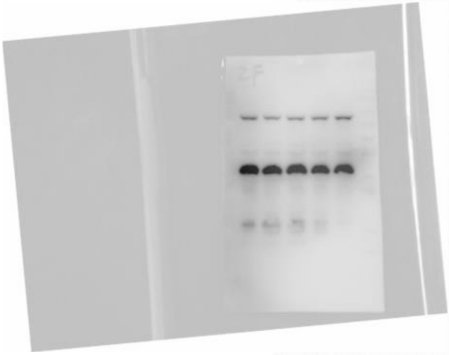

PI3K

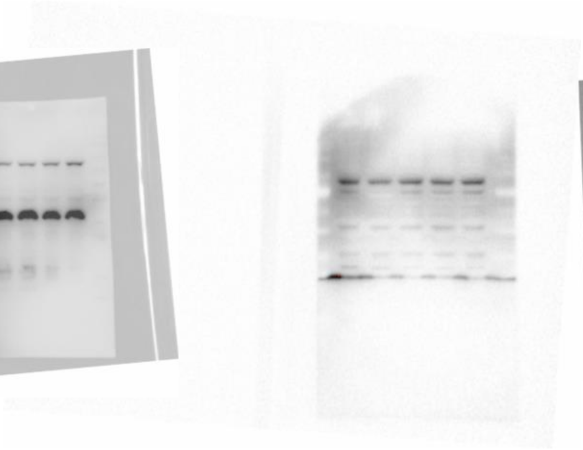

p-PI3K

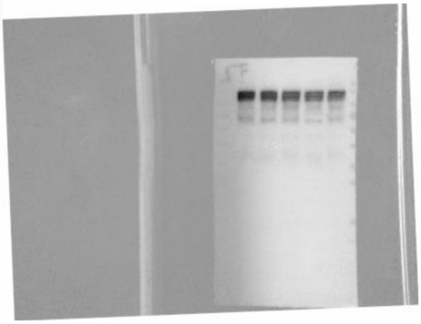

p-Syk

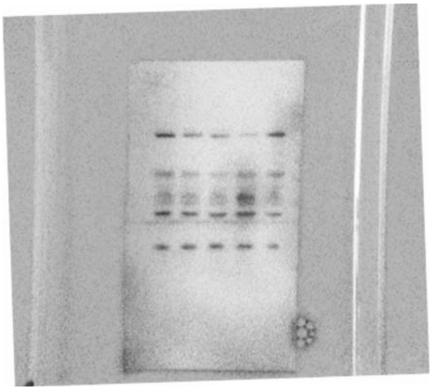

Syk

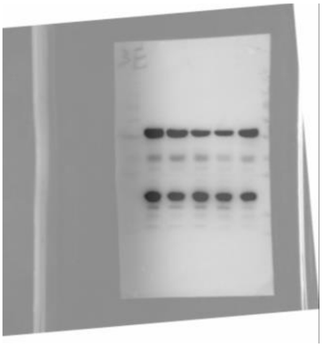

TREM2

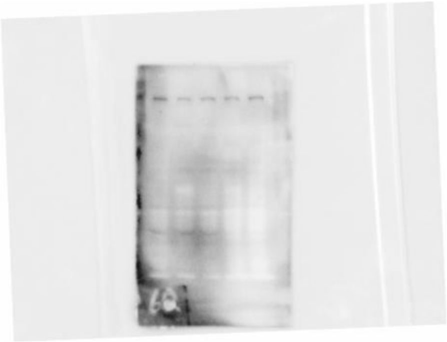

mTOR
